# Knowledge-guided docking: accurate prospective prediction of bound configurations of novel ligands using Surflex-Dock

**DOI:** 10.1007/s10822-015-9846-3

**Published:** 2015-05-05

**Authors:** Ann E. Cleves, Ajay N. Jain

**Affiliations:** Helen Diller Family Comprehensive Cancer Center, University of California, San Francisco, CA USA; Department of Bioengineering and Therapeutic Sciences, University of California, San Francisco, CA USA

**Keywords:** Docking, Protein flexibility, Surflex, Molecular similarity, Data fusion, Pose prediction

## Abstract

Prediction of the bound configuration of small-molecule ligands that differ substantially from the cognate ligand of a protein co-crystal structure is much more challenging than re-docking the cognate ligand. Success rates for cross-docking in the range of 20–30 % are common. We present an approach that uses structural information known prior to a particular cutoff-date to make predictions on ligands whose bounds structures were determined *later*. The knowledge-guided docking protocol was tested on a set of ten protein targets using a total of 949 ligands. The benchmark data set, called PINC (“PINC Is Not Cognate”), is publicly available. Protein pocket similarity was used to choose representative structures for ensemble-docking. The docking protocol made use of known ligand poses prior to the cutoff-date, both to help guide the configurational search and to adjust the rank of predicted poses. Overall, the top-scoring pose family was correct over 60 % of the time, with the top-two pose families approaching a 75 % success rate. Correct poses among all those predicted were identified nearly 90 % of the time. The largest improvements came from the use of molecular similarity to improve ligand pose rankings and the strategy for identifying representative protein structures. With the exception of a single outlier target, the knowledge-guided docking protocol produced results matching the quality of cognate-ligand re-docking, but it did so on a very challenging temporally-segregated cross-docking benchmark.

## Introduction

Docking of small molecules to protein binding sites by computational means is now a mature field, having been established on rigid ligands in the 1980s [1]. The first practical methods that addressed ligand flexibility in an automatic fashion appeared in the 1990s, with AutoDock [2], GOLD [3, 4], Hammerhead [5–7], and FlexX [8, 9]. These early reports shared a common validation strategy: re-docking of ligands into their cognate protein binding pockets, with success rates typically defined as symmetry-corrected RMSD ≤2.0 Å. On benchmark sets containing dozens of diverse targets, success rates in the late 1990s to early 2000s for top-scoring pose prediction were roughly 70–80 % [4, 10, 11]. Success rates at this level for cognate ligand re-docking have persisted across different data sets [4, 12], though lower success rates (closer to 60 %) have been reported for particularly challenging cognate-docking benchmarks [13, 14]. Assessment of cognate re-docking has continued, with a recent ACS symposium showcasing results for eight methods [15–22]. Among the more widely used methods (DOCK, FlexX, Glide, GOLD, and Surflex-Dock), using agnostic procedures for complex preparation that favored no method in particular, success rates in the low to mid 70 % range were typical.

Multiple groups showed that nominal success rates could be improved by ten to 20 % points through manipulation of the starting conditions for proteins and ligands. This is an inherent problem in this type of exercise because the construction of the problem embeds the correct answer, and knowledge of that answer can be used to bias results. The risks of employing such techniques in methodological validation [11] have been documented previously [23, 24].

Real-world practitioners of docking face entirely different problems, typically involving prediction of the binding modes of ligands during a design process, understanding the binding mode of a newly reported chemical series, or identification of new lead compounds through virtual screening. Binding-mode prediction is the focus of this study, and it can be important for visual modeling in compound design or as an adjunct to affinity prediction approaches, both simulation-based [25] and those that employ machine-learning [26, 27]. It is important to note that while the prediction of changes in binding affinity that result from minor changes to a ligand is a challenging and important problem, binding modes are not often *drastically* altered in such cases. So, the challenge for docking is where a more significant structural change in a ligand is made than from a methyl to an ethyl substituent. In such cases, where novel ligands are to be docked (commonly called the “cross-docking” problem), protocols that parallel those used for cognate docking perform poorly.

Three particularly influential studies of the cross-docking problem were published in the 2006–2008 time-frame. Warren et al. [28] made an independent study of several docking programs, both for non-cognate pose prediction and for screening enrichment. The pose prediction aspect involved seven targets (averaging roughly 180 ligands per target), making use of a single protein structure as a representative for each target. In this study, an expert choice was made with respect to protein structure, and expert users for each docking program were used. Performance was highly target dependent, with average success rates across the seven targets among the better performing methods ranging from 20 to 35 % (but with standard deviations of roughly the same magnitude). However, performance was also highly method dependent. While any single method yielded relatively poor overall performance, for four of the seven targets, at least one of the docking methods yielded top-scoring pose prediction success rates of at least 50 %. Sutherland et al. [29] explored all-by-all cross-docking using a set of 249 ligands spanning eight targets using two docking algorithms. They performed exhaustive cross-docking (each ligand against every non-native structure), observing success rates ranging from 18 to 24 %. In contrast to the results reported by Warren et al., only in the case of one target and one docking algorithm was the success rate over 50 %. Verdonk et al. [30] took a different approach, considering a highly curated set of 85 protein ligand complexes (the “Astex Diverse Set”) and then asking how docking performance was affected by considering alternative protein conformations for the *same* set of ligands. For this test, only the issue of protein conformation was addressed, with the protonation and tautomeric states being defined in the same manner as was done optimally for the cognate protein-ligand complex. In this case, the decrease in performance attributed to protein conformation variation was about 20 % points (from 80 to 61 %).

The latter two studies also considered the effects of using multiple protein variants for docking, each reporting improvements when effective selection strategies were adopted. Both observed that selection of structures as the targets for docking whose cognate ligand was similar to the non-cognate test ligand improved performance. Our own work [31], which made use of the challenging Sutherland data set, showed that agnostic selection of five protein structures improved overall cross-docking performance by roughly 20 % points over using a single-structure per target (from 26 to 45 % considering top-scoring poses without protein pocket optimization).

The more recent CSAR 2011–2012 Benchmark Exercise [32] largely confirmed the success rates observed in these previous studies for single-structure cross-docking. Multiple groups, using a diversity of docking methods, submitted pose predictions for four targets, where each was represented by a carefully chosen single protein structure. The ligand sets consisted of congeneric series: LpxC (3 test ligands), Urokinase (16 ligands, 1 series), Chk1 (38, 3 series), and Erk2 (39, 3 series). Percent correct over all tested docking methods at the 2.0 Å threshold for the two targets with multiple series was 28 % for Chk1 and 16 % for Erk2. For LpxC (with just 3 ligands), 75 % correct was reported, and for Urokinase the result was 57 %. Overall, for all methods against all targets, the likelihood of obtaining a correct pose as top-ranked was 29 %.

One aspect of the cross-docking problem that offers some reason for optimism is that the success rates for identification of a correct pose among all those produced by a particular method are much higher than those for the top-scoring pose [28, 29, 31, 32]. That is, the detailed ranking among a set of poses is often the point of failure, as opposed to a total failure to identify any reasonable solutions.

Clearly, the cross-docking problem is much more challenging than the cognate-docking assessments utilized during the infancy of the field. It has been clearly established that protein conformational variation plays an important role. Other aspects of binding site complementarity involving protonation or tautomerism also matter, but they have not been as carefully studied. Often discussed, but also not systematically studied, is the fact that an expert in a particular target system who has great facility with a particular docking method, can often obtain results that are far better than those obtained from naive naïve tool application to the same system. This appears to stem from knowledge of the target system (e.g. common variant configurations of a binding pocket), ways in which ligands tend to bind (e.g. particular recognition motifs that are relatively invariant), and the ability to guide a docking algorithm based on such knowledge.

**Fig. 1:**
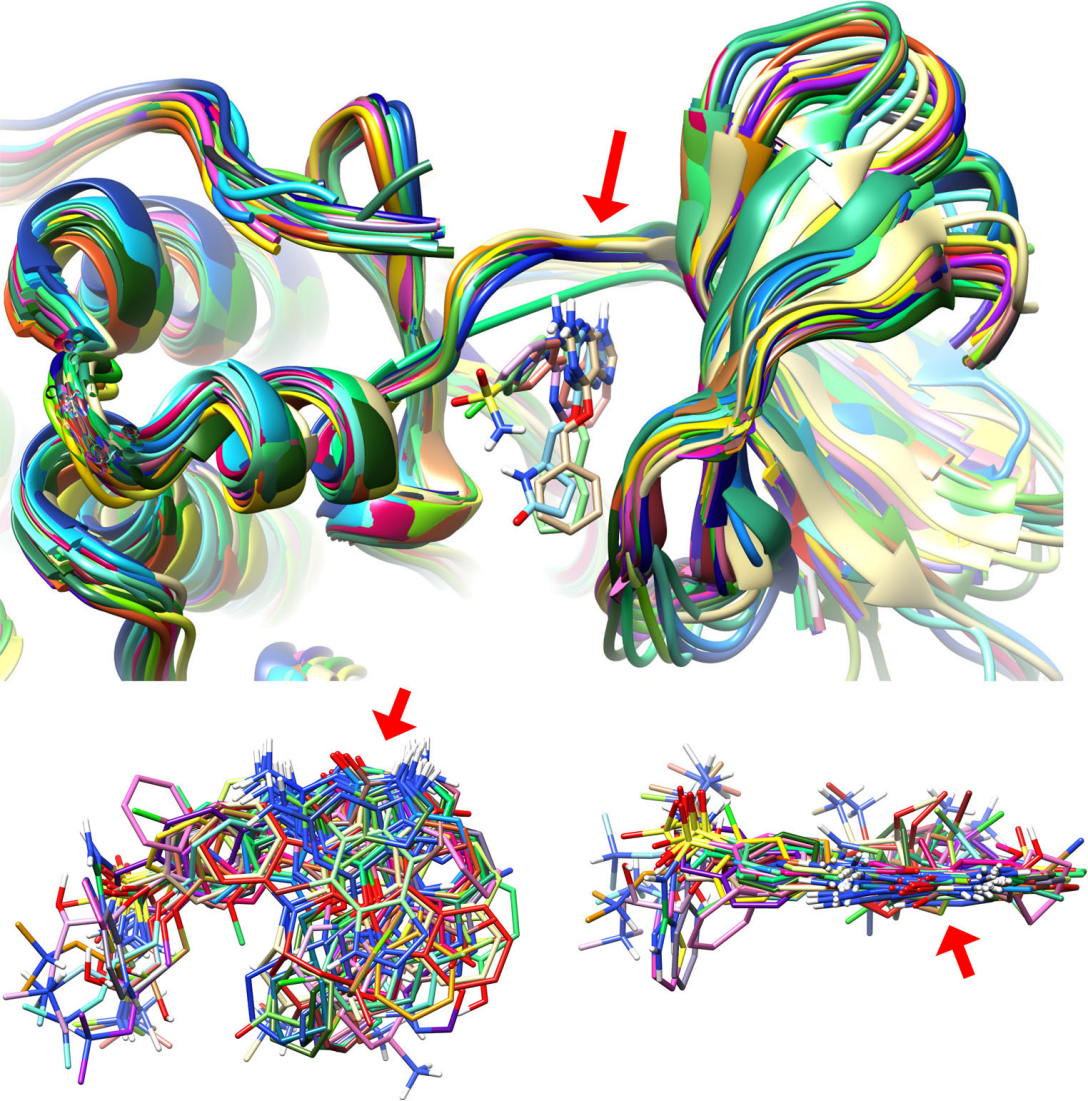
The structural information available about CDK2 inhibitors prior to July 2003: all 42 protein structures shown with five small ligands in the active site (*top*); all 42 ligands oriented with their hinge-binding moieties upward (*bottom left*); and all 42 ligands with the viewpoint from the kinase hinge (*bottom right*)

Figure 1 shows a snapshot in time of what was known about CDK2 inhibition using X-ray crystallography as of June, 2003. These represent the *earliest* one-quarter of CDK2 structures with active-site ligands that were deposited in the PDB as of Fall 2012. One can see the characteristic, and largely invariant, hinge binding motif of the ligands (red arrows). Faced with a question about binding mode, a new ligand may look little like those that have been studied experimentally. Choices must be made about which protein structures to use, how they should be prepared, how the binding site should be scoped and defined, and how (if at all) to make use of knowledge of other ligands’ binding modes to either guide or constrain the docking process. Fewer than half of the ligands whose bound structures were deposited in the PDB after those shown in Fig. 1 had significant 2D similarity to the initial set of 42 early CDK2 structures. Given that there was interest in experimental structure determination for the ligands bound to CDK2, it is reasonable to expect that, in many cases, there was uncertainty about the mode of binding, interaction with the protein, and whether significant conformational changes in the protein pocket were induced.

**Fig. 2:**
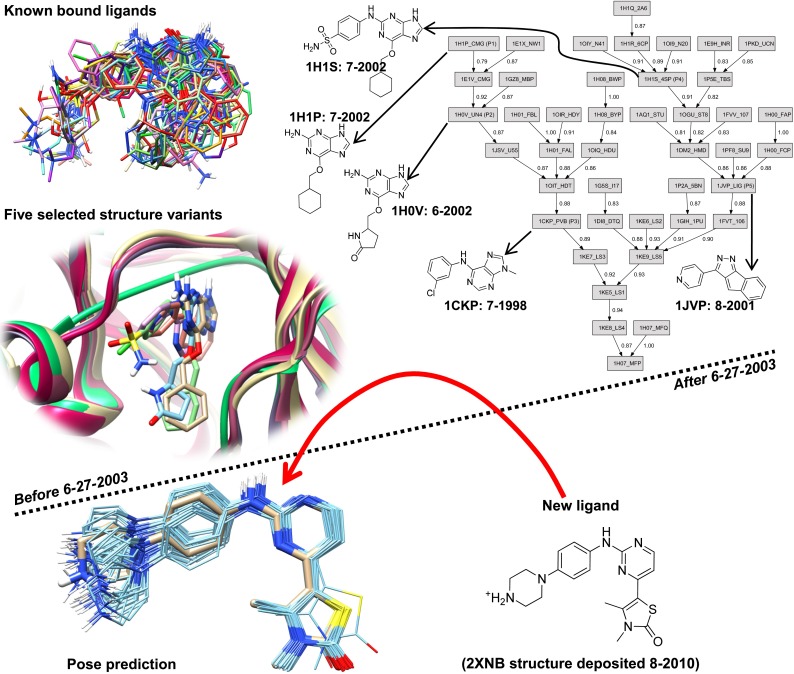
Temporal partitioning for cross-docking prediction: the information above the dotted line became publicly available prior to 6-27-2003 and is to be used to make predictions on ligands whose bound configurations was determined later

We believe that the most relevant and challenging question one can ask about binding mode prediction is whether one can, given information available at a particular time, make accurate predictions on ligands whose bound structure was determined in the *future*. Figure 2 illustrates this conceptualization of the cross-docking problem. For CDK2, the structural information includes 42 protein-ligand complexes for use in making predictions on 127 that were determined later in time. By constructing the task through temporal partitioning and by making use of public PDB structural data, the challenge is more difficult and realistic in two critical ways. First, the structural diversity seen in the test ligands compared with the knowns is high, because it is rare to see effort invested in determining a bound structure for a simple analog of another ligand whose bound structure was known long ago. The structural diversity *among* the test ligands is also high (for the same reason, with the exception of multiple structures from single studies). Second, restricting information to that available prior to a particular time removes a primary source of positive bias in modeling studies. The prediction task is not contaminated by knowledge that was gained by knowing the answer for the prediction at hand.

Methodologically, we describe and evaluate three new techniques (all procedures will be described in more detail in the “Methods, data, and computational protocols” section):

1. *Automatic protein structure selection* Given a collection of protein structures, there will be redundancy among many variants, and there will also be outliers. In order to perform well with respect to docking new ligands, structures must be chosen to be representative of the important variants in the collection. The procedure we have adopted computes all pairwise distances between structures based on protein binding pocket similarity [33, 34]. Given these data, choosing a specified number K is accomplished using K-means clustering, with the exemplars for each cluster being those that, on average, are closest to all cluster members (essentially the cluster centers). A mutual alignment is constructed using single-linkage hierarchical agglomerative clustering. Figure 2 shows the alignment tree along with the five chosen variants for CDK2.
2. *Dynamic use of known bound ligands to guide molecular alignment* In Fig. 2, the core scaffold (2-anilino-pyrimidine) of the new ligand can, for example, be found in the known 1H1S ligand (an anilino-purine). In this case, the common substructural fragment offers an excellent guide as to the correct primary alignment of the test ligand. Non-exhaustive dynamic substructural matching of a test ligand to the full set of previously known bound ligands is done in order to identify well-positioned fragments. These fragments are used to provide additional search focus on binding motifs that are supported by experimental evidence.
3. *Fusion of molecular similarity with docking scores to improve pose family ranking* The experimentally determined configuration of the 2XNB ligand (tan sticks, lower left of Fig. 2) manifests a core hinge-binding interaction common among many early CDK2 inhibitors. Clues as to the likely positions and orientations of the pendant groups may be found among these inhibitors as well (e.g. favorable positions for cations to make salt-bridges). In this work, the 3D similarity is computed for each predicted ligand pose from docking to the set of those previously known. These similarity values are re-cast as probabilities, and the natural relationship between probabilities and energies is exploited in order to provide a correction to the energetic score for each pose. Using the new scores, pose families are generated. In this example, the cluster of predicted poses covers the one derived from experiment, with the closest individual pose being 0.5 Å RMSD.

These new methods for addressing the cross-docking problem are implemented within Surflex-Dock, but the concepts are general and should be of broad utility. We present results on a newly curated cross-docking benchmark, which we believe to be the largest and most relevant that is publicly available. There are 949 test ligands, spread across ten extensively studied pharmaceutically relevant targets (ordered from most to least test ligands): carbonic anhydrase II (CA-II), cyclin-dependent kinase 2 (CDK2), HIV protease (HIV-PR), thrombin, beta secretase 1 (BACE1), $\hbox {HSP90}\alpha$, map kinase 14 (MAPK14), $\hbox {PPAR}\gamma$, protein tyrosine phosphatase 1b (PTP1b), and the non-nucleoside site of HIV reverse transcriptase (HIV-RT). At the time of curation in Fall 2012, these were among the most heavily represented targets within the PDB in terms of non-covalently bound ligands, with the particular selections made to avoid redundancy or lack of pharmaceutical relevance. For each target, all variants were identified by UniProt annotation, and partitioning was done based on PDB deposition date, with the earliest 25 % of structures forming the known set from which all predictions would be made and the subsequent 75 % forming the test ligands. The benchmark data set is called PINC (“PINC Is Not Cognate”). See www.jainlab.org for details on PINC availability.

By combining these new techniques with our previously established multi-structure docking protocol, we achieved a mean success rate for top-scoring pose families of over 60 %, with the three most challenging targets from our previous study (CDK2, MAPK14, and thrombin [31]) yielding mean performance of 71 %. Considering the top two pose families, the overall success rate was 74 % (80 % for the aformentioned trio). Among all pose families returned, the success rates were, respectively, 88 and 90 %. In this study, the most challenging targets were HIV-PR, PTP1b, and $\hbox {PPAR}\gamma$. These shared in common the highest proportion of test ligands whose structures were not only very different by 2D similarity to previously known ligands, but they were also very different in terms of 3D similarity (in their experimentally determined bound poses) to previous ligands. Other aspects such as ligand flexibility and binding site volume were less important.

These results are comparable to the best results obtained in difficult *cognate* docking benchmarks [13, 14, 23], though they are not quite as good as the best results obtained on the Astex Diverse Set [12, 15], a carefully curated “clean” cognate benchmark upon which many methods exhibit high performance. In a practical sense though, for targets where there is significant knowledge to be exploited, the methods presented here offer an automated means to deliver robust performance in a wide variety of cases, with expectations of the correct binding mode being among the top 2 predicted pose families over 70 % of the time, among the top 5 over 80 % of the time, and being among all those predicted 90 % of the time. For an automatic approach, not requiring a human expert, this represents a significant improvement in binding mode prediction.

In a more general sense, the new techniques presented here should be applicable beyond the particular implementation described. We believe that hybrid approaches that combine information from docking and scoring, ligand similarity, and protein pocket similarity will frequently show synergistic performance improvements for lead discovery and for predictions of binding mode, affinity, and off-target biological effects.

## Methods, data, and computational protocols

The results of this study are derived from a new benchmark data set for cross-docking, which will be described in detail first. Three new methods for making effective use of structural information, about both proteins and ligands, will be described next. Readers may find the details of the computational protocols for data set preparation, docking, and evaluation (that end this section) of more interest after having read the Results and Discussion.

### Structural data: the PINC benchmark

A query of the Protein Data Bank [35] on 8-1-2012, seeking X-ray protein structures containing bound ligands, produced an initial set of complexes. These were filtered to retain ligands that were non-covalently bound, contained only atoms including [H C N O S P F Cl Br I], and had molecular weight less than 1000. Ligands were rejected that lacked at least three heavy atoms whose minimal distance (van der Waals surface-to-surface) with the protein was less than 1.0 Å. Further, the relationship of the ligands to the sites was assessed to eliminate ligands just grazing a protein’s surface, a notion of “buried-ness” described in Spitzer et al. [34], which assesses a ratio of the number of nearby protein atoms relative to the total number of ligand heavy atoms. The filtering criteria were designed in order to preserve all of the ligand binding sites in the Astex Diverse cognate-docking benchmark of 85 complexes [12]. Note that in order to obtain as many examples as possible, no limit was set on crystal structure resolution or other technical aspects of structure quality.

The resulting set contained 30,999 PDB structures, corresponding to 63,699 liganded binding sites. These were organized by annotated UniProt ID and sorted by the number of ligands per target. The top 16 such targets each contained more than 70 ligands, and 10 of these targets form the benchmark data set summarized in Table 1. These ten targets represent a diverse set of protein types: a tyrosine phosphatase (PTP1b), two aspartyl proteases (BACE1 and HIV-PR), a mitogen-activated protein kinase (MAPK14), a serine-threonine kinase (CDK2), a serine protease (thrombin), a ligand-modulated transcription factor ($\hbox {PPAR}\gamma$), a metal-dependent dehydratase (CA-II), a heat-shock protein (HSP90), and a transcriptase (HIV-RT). All are either targets of existing drugs or have been actively pursued as drug targets. Three of the 16 were eliminated on the basis of redundancy with another target or target class (P00760: trypsin, P00742: factor Xa, O14757: chk1). Another three were eliminated because the proteins were from non-human, non-pathogenic organisms. These three were judged to be of limited pharmaceutical relevance (P00489: glycogen phosphorylase [rabbit], P19491: glutamate receptor 2 [rat], and P29476: nitric oxide synthase [rat]). Additional details on preparation protocols can be found below in Computational Procedures.

**Table 1:** Summary of target and ligand characteristics, sorted by binding site volume

Target	UniProt	N early	Date cutoff	N future	Volume (Å$^3$)	MW (mean ± SD)	NRot (mean ± SD)
PTP1b	P18031	17	15-Jan-03	52	3102	505 ± 151	10 ± 5
BACE1	P56817	34	4-Dec-07	103	2360	481 ± 154	10 ± 7
MAPK14	Q16539	30	12-Oct-07	92	2232	435 ± 87	7 ± 3
HIV-PR	P0336[6/7/9]	42	^a^	127	1892	622 ± 121	16 ± 5
Thrombin	P00734	37	26-Sep-01	114	1858	430 ± 97	9 ± 4
$\hbox {PPAR}\gamma$	P37231	21	22-Aug-06	62	1443	414 ± 122	8 ± 4
CA-II	P00918	42	21-Apr-05	128	1420	299 ± 81	6 ± 3
$\hbox {HSP90}\alpha$	P07900	32	28-Jun-07	98	1329	301 ± 111	4 ± 3
CDK2	P24941	42	26-Jun-03	127	1237	343 ± 78	5 ± 2
HIV-RT	P04585	15	19-Jul-99	46	614	369 ± 79	5 ± 2

^a^HIV-PR was annotated in the PDB within three UniProt families, treated each with their own date cutoffs, respectively: 22-Dec-05, 11-Apr-97, and 11-Jun-02

The total number of ligand binding sites was 1261. Overall, 89 % of the structures had resolution of 2.5 Å or better, and the poorest resolution structure was 3.3 Å. Data for each target was partitioned by sorting on the PDB deposition dates, making use of the *oldest* 25 % (312 ligands) for use as information to guide binding mode predictions and reserving the remaining 75 % (949 ligands) for testing docking protocols. Table 1 breaks the data set down by target, sorted by binding site volume. HIV-PR had the largest and most flexible ligands, with the other pole being occupied by $\hbox {HSP90}\alpha$ and CA-II. Overall, 18 % of test ligands had 3 or fewer rotatable bonds, 38 % from 4 to 7, 24 % from 8 to 12, 16 % from 13 to 19, and 5 % had 20 or greater.

**Fig. 3:**
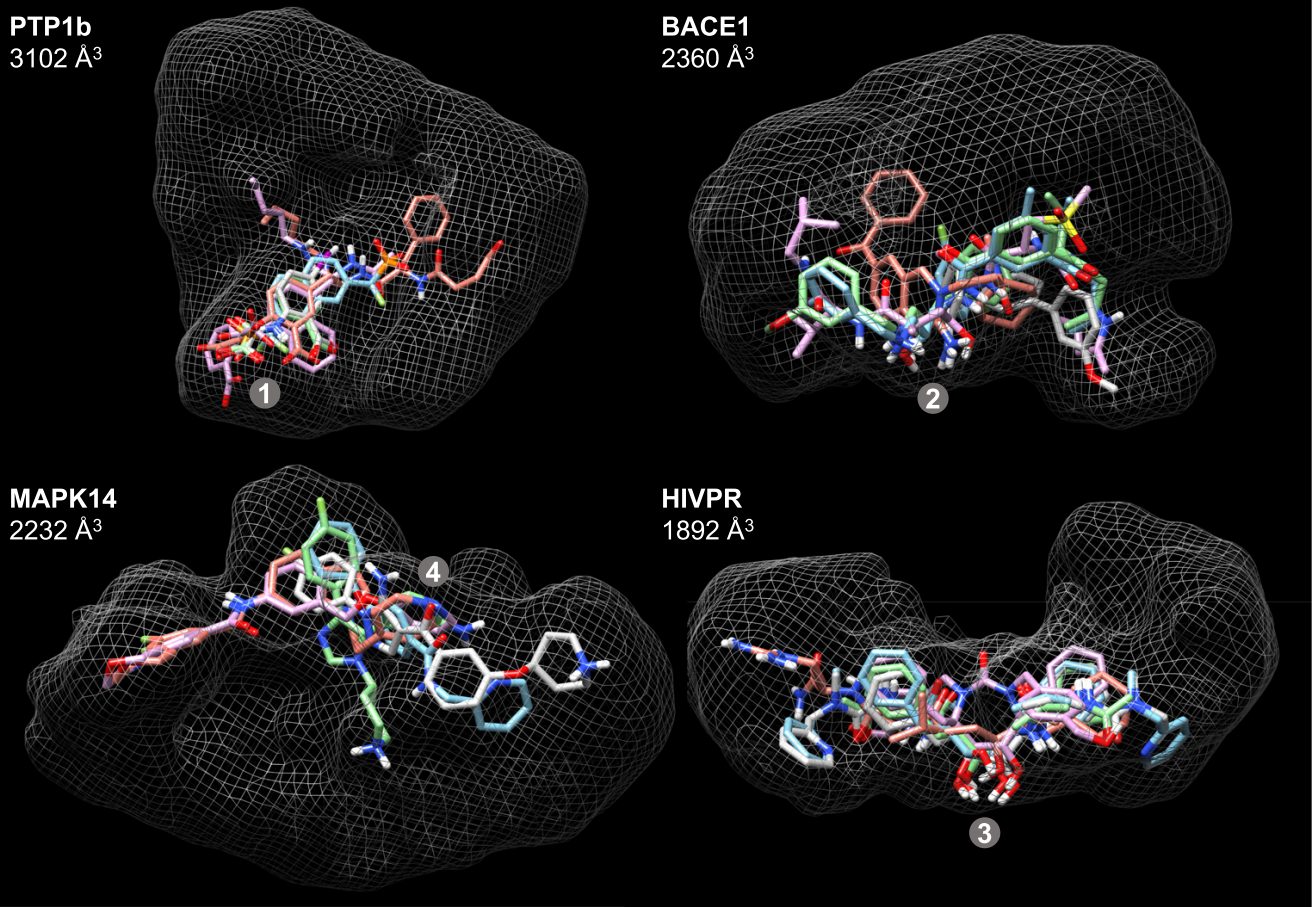
The four binding sites with the largest volumes

**Fig. 4:**
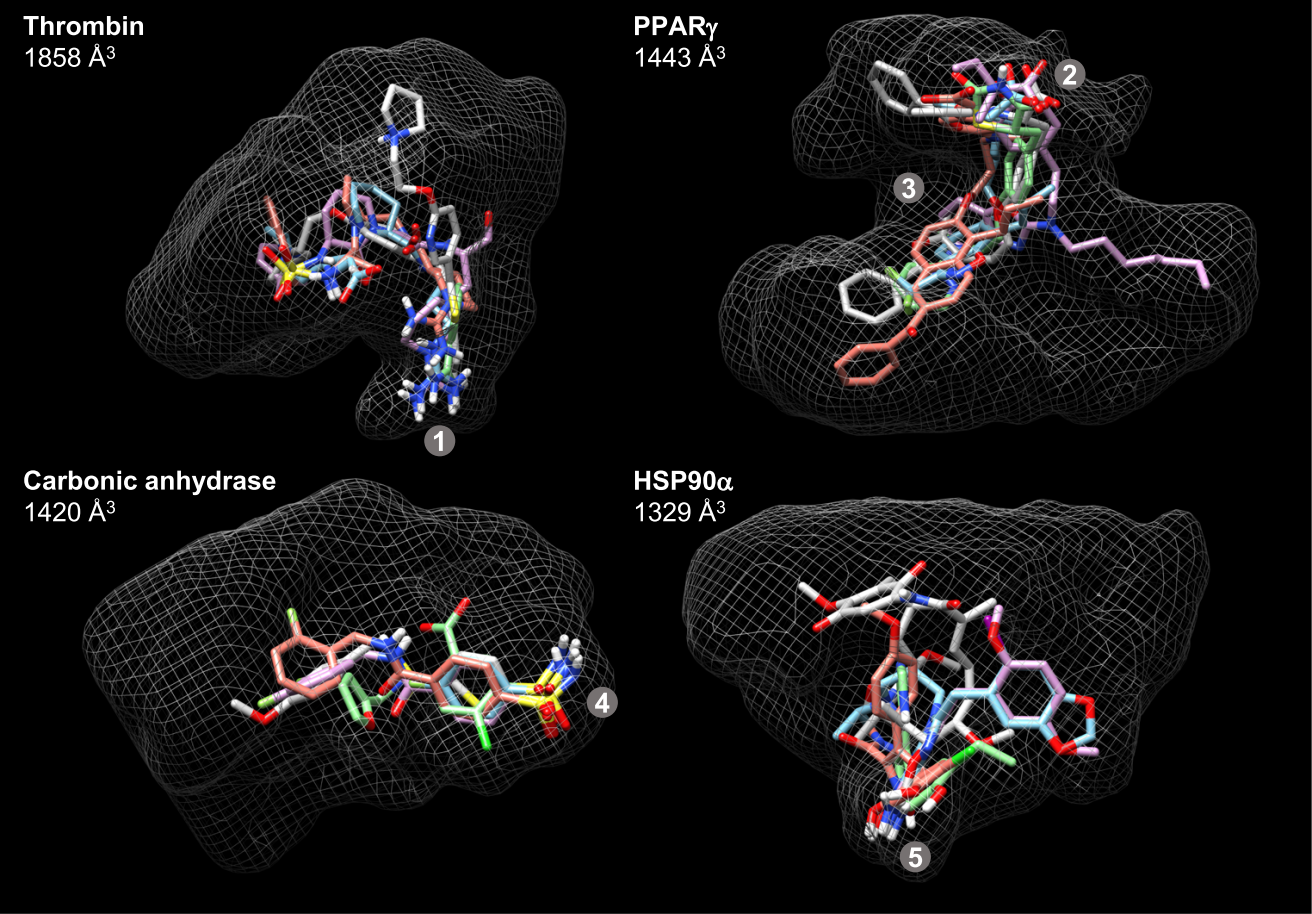
The protein binding sites with volume sizes ranked 5–8

The eight largest binding sites are depicted in Figs. 3 and 4. Each is shown with white mesh enveloping the full scope of the binding site as explored by the test ligands. Within each target site, five ligands are shown, those examples belonging to the binding sites chosen as being representative of each target’s *early* set (the choice process is described below).

The four largest sites had volumes spanning 2000–3000 Å$^3$. PTP1b is the largest, by far, with a characteristic ligand binding mode that involves a salt-bridge typically between a carboxylate on the ligands and Arg-221 on the protein (marked as **1** in Fig. 3). The site can accommodate extremely large ligands, including some that span the longest extents of the envelope depicted. BACE1 is an aspartyl protease, where the common recognition motif of inhibitors includes interaction with the active-site aspartic acid residues (**2** in the figure). HIVPR, another aspartyl protease, is analogously marked (**3** in the figure). MAPK14 has its hinge binding region marked (**4**).

In Fig. 4, thrombin has the S1 binding pocket marked (**1**), with ligands typically containing basic moieties for salt-bridging to an aspartic acid residue, but neutrally charged S1 elements emerged over time in development of thrombin inhibitors. $\hbox {PPAR}\gamma$ has a complex active site, with a site frequently occupied by a ligand carboxylate (**2**) and a helix (**3**) around which the typically very flexible ligands bend. The $\hbox {PPAR}\gamma$ ligands in Fig. 4 all exhibit a canonical binding mode, but a very different alternate mode was discovered over time. CA-II ligands interact with an essential zinc ion (marked **4**), very often containing a sulfonamide that is thought to interact in its anionic form [36]. The active site opening is fairly wide, offering opportunities for many different protein-ligand interactions. HSP90 is marked with a characteristic interaction to residues Asp-93 and Ser-52 (**5**).

For all ten targets, the overall volume that is explored by the test ligands is much larger than that observed within the structures used for docking. Even considering the full complement of the “early quarter” of protein-ligand complexes for each target, the scope and variety of interactions observed later in time represent very challenging problems for binding mode prediction. For this reason, an effective strategy for choosing representative protein structures from among those available at a particular time, independent of *any* knowledge of the future ligands to be predicted, is critical.

### Systematic choice of representative protein structures

Given a collection of protein structures, there will be redundancy among many variants, and there will also be outliers. In order to perform well with respect to docking new ligands, structures must be chosen to be representative of the important variants in the collection. The procedure we have adopted computes all pairwise distances between structures based on protein binding pocket similarity, using the PSIM approach [33, 34]. Given these data, choosing a specified number K is accomplished using K-means clustering, with the exemplars for each cluster being those that, on average, are closest to all cluster members (essentially the cluster centers).

A mutual alignment is constructed using single-linkage hierarchical agglomerative clustering. Figure 2 shows the alignment tree along with the five chosen variants for CDK2. Note that the five chosen representatives (the protein ensemble) come from different parts of the tree. With the exception of 1H1P, all are clearly part of groups of closely related protein variants (the values on the graph’s edges are protein pocket similarity scores). The protein alignment is done to maximize the surface concordance of the protein variants according to the PSIM metric.

### Exploitation of known substructural fragments

**Fig. 5:**
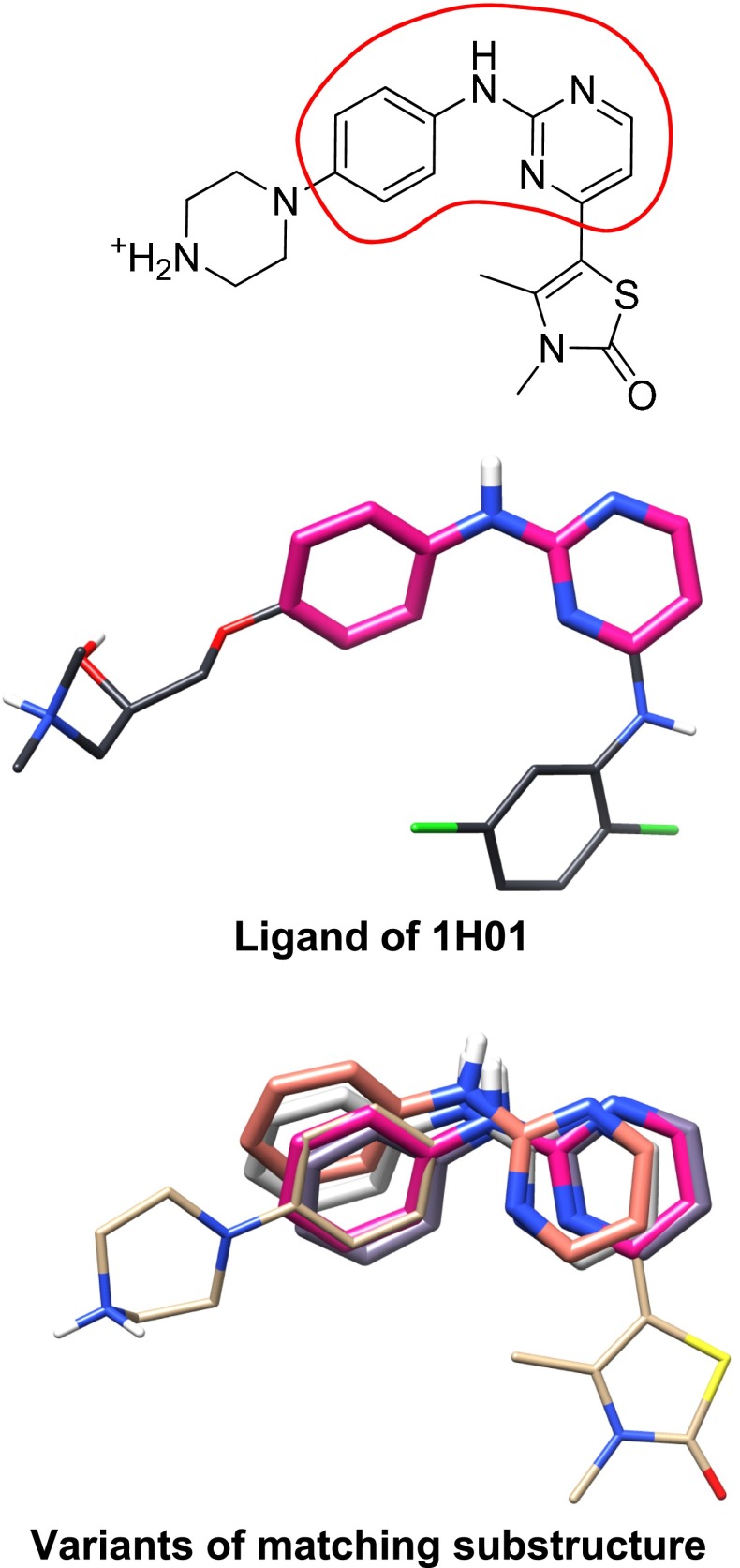
Dynamic substructure matching of a ligand to be docked to ligands whose bound poses are known is used to provide additional search focus on known binding motifs. Note that the ligand conformations shown are from the respective crystal structures to illustrate the relative alignments of known fragments to those present in the subject ligand

In Fig. 5, the core scaffold (2-anilino-pyrimidine) of the ligand in question can, for example, be found in the ligand of 1H01 (among several others). In this case, the common substructural fragment offers an excellent guide as to the correct primary alignment of the test ligand. Non-exhaustive dynamic substructural matching of a test ligand to the full set of previously known bound ligands is done in order to identify well-positioned fragments. These fragments are used to provide additional search focus on binding motifs that are supported by experimental evidence.

For each molecule to be docked, a set of substructural matches (containing at least four heavy atoms) are identified from the set of bound ligands. The search for matching substructures is performed in a depth-first manner, while keeping track of the largest yet-discovered substructure and the total number of recurrence initiations. The search is terminated either by exhaustion or when more than 10,000 recurrence initiations have occurred since the last discovery of a larger matching substructure between the molecule to be docked and the known molecule. Redundant matches are eliminated for each known molecule. After identifying all such substructural matches for all of the user-provided molecules, the GSIM 2D similarity score [37] is computed between each substructural match and the whole ligand, with the top scoring matches being retained (with a default maximum of 50 matches for docking a particular ligand).

The substructural matches are used *in addition* to the standard alignment procedures within the Surflex-Dock search algorithm [10, 38]. The additional computational cost of this additional search focus is low, because the substructure identification procedure is non-exhaustive and computing a maximum of 50 additional alignments per ligand conformation is computationally inexpensive. The median docking times using a single computing core on a standard desktop workstation circa-2013 were roughly 5 min per ligand for targets with typically sized ligands (e.g. CDK2), but for targets with highly flexible ligands (e.g. HIV-PR), times increased to 10–20 min per ligand.

### Use of molecular similarity to influence pose ranking

The experimentally determined configuration of the 2XNB ligand (tan sticks, lower left of Fig. 2) manifests a core hinge-binding interaction common among many early CDK2 inhibitors. Clues as to the likely positions and orientations of the pendant groups may be found as well (e.g. favorable positions for cations to make salt-bridges). Here, the 3D similarity of each predicted ligand pose from docking to the set of those previously known is computed. These similarity values are re-cast as probabilities, and the natural relationship between probabilities and energies is exploited in order to provide a correction to the energetic score for each pose. Using the new scores, pose families are generated. In this example, the cluster of prediction poses covers that derived from experiment, with the closest being 0.5 Å RMSD.

**Fig. 6:**
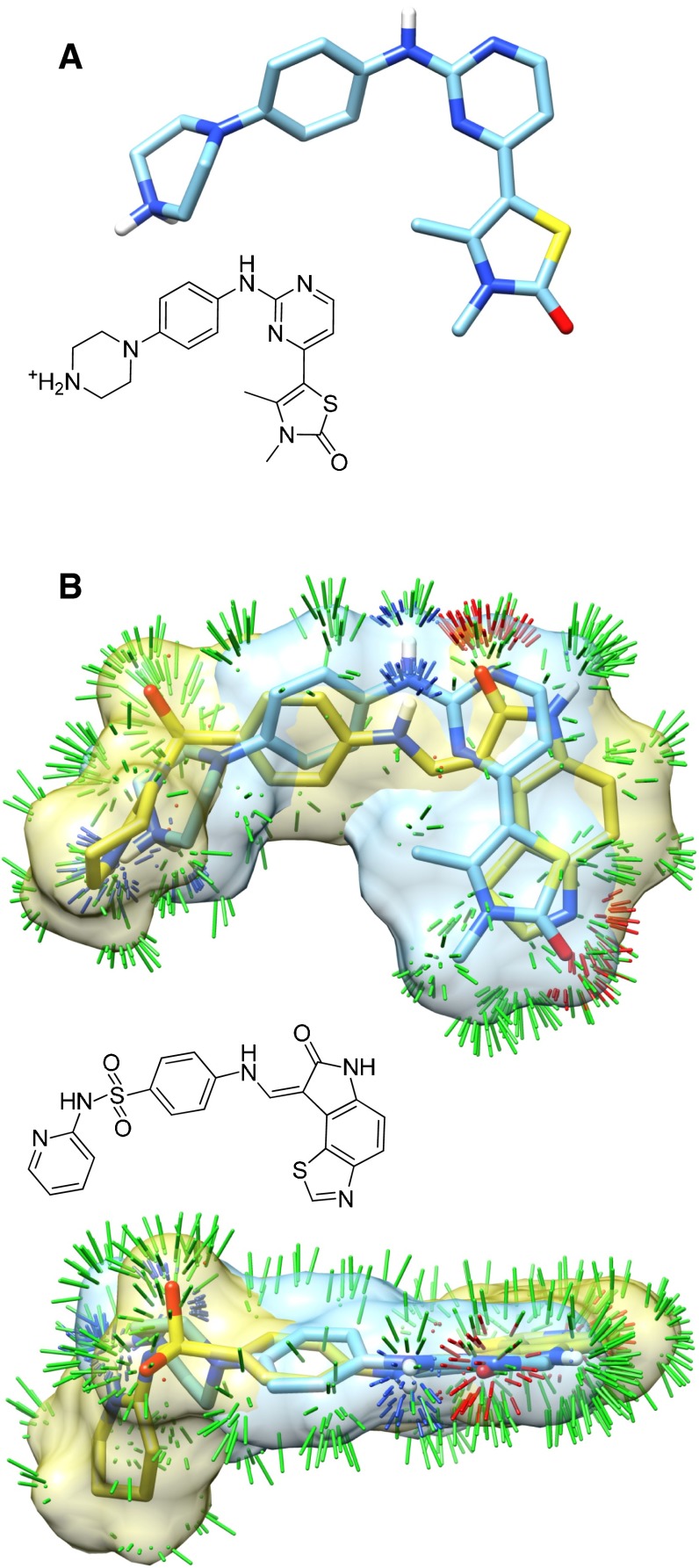
Molecular similarity to bound ligands of different scaffold types can help in pose-ranking

Figure 6 shows a predicted pose for the 2XNB ligand (A), along with a depiction of its surface shape and electrostatic similarity to the bound pose of the ligand of 1FVV (B). Green sticks indicate high shape similarity, and blue and red sticks depict high similarity in polar characteristics (positive and negative features, respectively). While both compounds are aniline derivatives, it is the similarity of the more structurally divergent parts of the molecules that drive the high similarity.

**Fig. 7:**
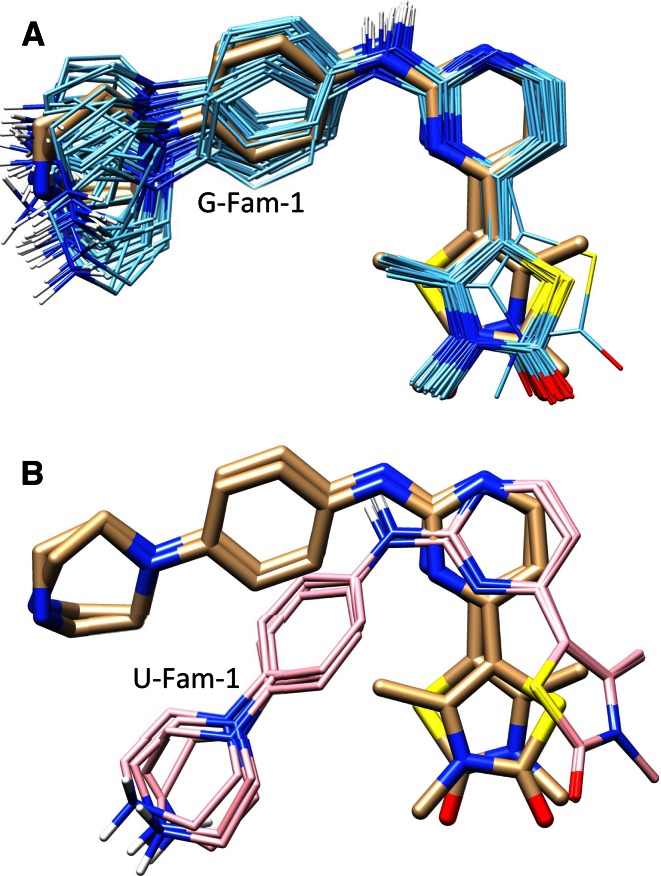
The top scoring pose families for the 2XNB ligand for knowledge-guided docking (**a** marked “G-”) and unguided docking (**b** marked “U-”). The crystallographic pose (two alternates) are shown in *thick tan sticks* with the docked pose families shown in contrasting color

We have previously introduced the idea of using *pose families* of closely-related ligand configurations to represent the results of docking [38]. Rather than treating the final predicted pool of $n$ poses as individual and independent predictions, pose families are constructed based on RMSD, and they are ranked based on Boltzmann-derived probability scores. A given score of $x$ (between 0 and 1) for a particular pose family means that it is expected for the experimentally observed bound configurations of the ligand in question to fall within that family with probability $x$. Figure 7 shows the two top-scoring pose families for the ligand of 2XNB. Without adjustment based on prior knowledge of other bound ligands, using only docking scores, the bottom (incorrect) pose family received a probability score of 0.57, with the top receiving a score of 0.31. The challenge is to make use of knowledge of prior ligand binding modes in order to distinguish the correct binding mode (A) from the incorrect mode (B).

The procedure we employ takes an idea from statistical potentials, which derive energy functions from observed distributions of molecular configurational properties (typically distances). In the case of amino-acid residues, the free-energy of interactions between residue types $i$ and $j$ is given as follows [39]:

1 $$\begin{aligned} w_{ij} = -R T \ln \left( \frac{\rho _{ij}(r)}{\rho ^*} \right) \end{aligned}$$

The notion is to determine the distribution of observed configurations compared with a reference state. Configurations that are common in the observed data relative to the reference state lead to a high relative likelihood and consequently a favorable negative energy. Here, we use this idea to provide an energetic correction predicted molecular poses, where those that appear to be more “native-like” are treated in an analogous fashion to amino-acid distances that are frequently observed within experimental data. We must compute a relative likelihood that a given pose for a ligand is native-like given information about the experimentally determined poses of other ligands.

Suppose we have a collection of docked poses for a ligand, denoted $L_{1\ldots n}$, some of which are close to native and some not. We can use a similar formulation to Eq. 1 by expressing the similarities of these poses to native poses in terms of probabilities. We have previously shown how to transform the results of molecular similarity computations into probability values by comparing the magnitude of a similarity score for molecule A versus B to the distribution of scores for A and B compared with a random background set of molecules [40, 41]. In that work, given the maximal similarity of A to B (in any energetically reasonable conformation of either molecule) the problem was to assign a probability of observing a similarity value or that magnitude or higher. In the present case, we have a different situation, in two respects. First, we have *particular* poses of a ligand $L_{1\ldots n}$ that fit within the active site of a particular protein; as such, they represent a small fraction of the configurational space available to the ligand. Second, we have a *set* of native poses of multiple active-site bound ligands, one each (denoted $K_{1\ldots m}$). We compute the mean and standard deviation of the distribution of Surflex-Sim 3D pairwise molecular similarities between the predicted poses $L_{1\ldots n}$ and the known bound poses $K_{1\ldots m}$ (denoted $\mu$ and $\sigma$).

So, we have estimated $\mu$ and $\sigma$ for the population of all poses of $L$ that fit within the active site, based on the results of the docking procedure. For a particular pose $L_i$, its *average* similarity to the *set* of bound poses is computed (similarity function denoted by $S$). Within the population of poses for $L$ that fit within the active site, the average of these $m$ similarities should have a distribution with mean $\mu$ and with variance $\sigma ^2/m$. We define a correction to the energy associated with pose $L_i$ as follows:

2 $$\begin{aligned} s_i&= \frac{1}{m} \sum \limits _{j=1}^m S(L_i,K_j) \end{aligned}$$

3 $$\begin{aligned} \sigma '&= \frac{\sigma }{\sqrt{m}} \end{aligned}$$

4 $$\begin{aligned} p_i&= 1 - \frac{1}{2}\left( 1 + erf\left( \frac{s_i-\mu }{\sigma ' \sqrt{2}} \right) \right) \end{aligned}$$

5 $$\begin{aligned} w_i&= -R T \ln \left( \frac{1}{p_i} \right) \end{aligned}$$

Equation 4 is simply the area under the right-hand side (high similarity) of the expected distribution of average similarity values to the known poses. A predicted ligand pose that looks much less native-like than other predicted poses would receive a low similarity score, resulting in a value close to 1 from Eq. 4 and an energetic correction of close to zero. Conversely, a predicted ligand pose that looks very native-like compared with other poses would receive a low probability and a large, favorable energy correction from Eq. 5. In practice, a lower bound of $1 \times 10^{-6}$ is used for the probability scores to limit the maximum size of the score adjustment.

Recall from Fig. 7 that the two top-scoring pose families for the ligand of 2XNB were incorrectly ranked, with the bottom (incorrect) pose family receiving a probability score of 0.57 and the top 0.31. After the similarity-based adjustment of docking scores using similarity to known, bound inhibitors, the probability scores changed to 0.0001 and 0.98, respectively. The value of the docking score adjustment for the single most native-like pose of the ligand was +3.1 (units of $pK_d$). The labels “G-Fam-1” and “U-Fam-1” indicate “knowledge-guided protocol family number 1” and “unguided protocol family number 1” respectively. This labeling convention will be used throughout the figures to identify protocols and pose family rank numbers.

### Computational protocols

Automatic procedures were used for protein and ligand preparation (including protonation and assignment of tautomeric states), test ligand pose randomization, binding site alignment within each target, and setup of all docking runs. Manual inspection of protein-ligand complexes for clear errors resulted in corrections for less than 5 % of structures. The full PINC benchmark along with the scripts used to produce the primary results of this study are available at www.jainlab.org.

#### Data set preparation

The pipeline for automated curation and alignment of the ligand-bound protein variants was described in a previous study that was focused on binding site comparison [34], and the overall strategy and characteristics are described above. Manual curation was required post facto to identify and correct ligand structural errors (typically bond order mistakes), and to identify cases where the automatic procedures yielded examples inappropriate for testing variations of docking procedures. Such cases included those where a metal ion is generally not present in a binding site (e.g. in thrombin and CDK2) but where one is required for ligand binding (e.g. zinc-dependent thrombin inhibitors and magnesium-dependent CDK2 ligands) and cases where the ligand was occupying a non-overlapping site. No attempt was made to “match” the test sites to the training sites in terms of protein configuration, protonation or tautomeric state, or ligand similarity or size. The only requirement was that ligands for a site within a particular target bound in roughly the same place (i.e. that the centroid of any particular ligand was not too far from the centroid of all of the ligands).

For each target, the collected curated structures (both training and testing) were subjected to all-by-all protein pocket alignment (using Surflex-Dock’s *psim_align_all* command). The command produces pairwise alignments (similarity scores and corresponding transformations) along with (potentially multiple) single-linkage hierarchical protein-similarity trees, where the alignments within each tree are from the “descendant” protein to it’s “parent.” Using the PDB deposition dates, the earliest 25 % of complexes were partitioned from the remaining complexes, which were used for testing. Five exemplars for each target were chosen from the early complex set using Surflex-Dock’s *psim_choose_k* command.

Given the five identified cluster centers, the early complexes in their mutual alignment, and the testing complexes in the same mutual alignment, each target’s docking data was derived. For each target, this consisted of the following: protein[1-5].mol2 (protein active sites trimmed around the bound ligand), ligand[1-5].mol2 (the bound ligands), EarlyHints.mol2 (all ligands from the early complexes), TestRef.mol2 (the test ligands in their bound poses), and TestMols.mol2 (the test ligands as input to the docking process). The test ligands were assigned random torsional angles for all rotatable bonds, then they were minimized, and their alignment parameters were assigned random values. This procedure was employed in order to remove bias and memory effects from the input to the docking protocols. Preparation scripts were also produced to build the input files for docking, including the “protomols” used within the Surflex-Dock algorithm.

Every effort has been made to produce a clean benchmark, but the challenges in constructing large-scale sets from the PDB should not be underestimated. The single largest challenge is the variable quality of information about bound ligands. For example, the ligand of 2XNB (see Fig. 2) was incorrectly represented within the PDB as an extremely high-energy tautomer of the correct structure (the single-bond between the aniline nitrogen and phenyl ring is swapped with the adjacent double-bond in the aromatic ring). We employ heuristic computational procedures to identify and correct many such mistakes, but some are detectable only by human inspection. Manual inspection and correction affected less than 5 % of the complexes.

#### Docking protocols

Within the docking procedure itself, two variations were tested, one using substructural hints (see Fig. 5) and one without. The former was specified by adding the option *-lmatch EarlyHints.mol2* to the docking command *gdock_list*, and the latter omitted the option. Following the docking procedure, two variations of pose-family generated were tested, one making use of bound ligand poses (see Fig. 6) and one without. The former was specified by adding the option *-posehints EarlyHints.mol2* to the *posefam* command, and the latter omitted the option. Additional experiments to examine the effect of protein variant choices were done by using alternate target input specifications, but all other aspects of the computations remained the same.

The docking procedure employed was a variation of the ensemble-docking protocol implemented within Surflex-Dock and reported previously [38]. The previous ensemble docking command of Surflex-Dock has been generalized for this work in order to support multiple strategies for controlling the optimization of ligand poses, but the scoring function was unchanged. The generalized version makes use of pre-searched conformations of ligands to be docked. This procedure enumerates reasonable, diverse, low-energy conformations (including flexible ring variations) of a given input ligand (by default, up to 200 conformations are retained). Ligand preparation for docking was made using the *search_library* command of Surflex-Dock. Note that all ligand preparation for docking was done beginning from ligand coordinates with no “memory” of the crystallographic coordinates (see the description of the preparation of TestMols.mol2 above).

The standard command employed for the docking runs using substructural hints was: *surflex-dock.exe-lmatch EarlyHints.mol2-pgeom gdock_list Mols/pre-list Targets loghints*. The standard command for producing pose families using bound ligand poses was: *surflex-dock.exe-posehints EarlyHints.mol2 posefam loghints*. Docking was performed using Surflex-Dock version 2.742.

#### Evaluation of results

The pose families resulting from docking under the different protocols were evaluated by computing, for each test ligand, the minimum RMSD for each family to the reference pose of the ligand, correcting for internal symmetries. In the case of HIVPR, C2-symmetry was also accounted for because of the geometry of the active-site. The bulk of analysis involves consideration of the cumulative histogram of RMS deviations on a per-target basis. The cumulative histogram is simply a numerical integration over a standard histogram which transforms the frequency count (ordinate) for each binned value (abscissa) into a cumulative proportion (range 0–1, ordinate) for each value (abscissa).

In order to assess the degree of variation among protein variants for each target, the global distribution of all within-target protein similarity values was used to set a threshold, below which protein variants were considered to be novel. In an analogous fashion, binding modes for test ligands were judged for novelty based on their maximal similarity to previously known bound ligands (both in their experimental poses). The threshold for ligand binding mode novelty was also based on a global analysis for all targets.

In some of the analyses, 2D molecular similarity was employed to assess the degree to which a test ligand was closely related to previously known ligands. The GSIM method was employed in all such analyses [40]. Given two molecules A and B as input, the method identifies all subgraphs of molecule A up to depth 3 at each heavy atom. For each subgraph, its existence is checked in molecule B. The tally of matches is kept, weighted to favor subgraphs rooted at heteroatoms. The computation is carried out bi-directionally, and the result normalized to a scale of 0–1.

## Results and discussion

The prediction task examined here parallels that frequently seen by molecular modelers: given some new molecule that is structurally different from those seen before, identify the manner in which it binds the particular active site in question. Questions about binding mode frequently occur following verification of biological assay against a target of interest following a high-throughput screen. They also arise when new ligand structures are reported within the scientific or patent literature. Understanding the geometric relationship between new ligands and ones that have been studied can be of great utility in shaping design decisions during lead optimization. By partitioning our set of protein-ligand complex data temporally, by target, we have tried to mirror the interesting case: one where there is sufficient uncertainty about the binding mode of a particular ligand (or its effect on a binding pocket) that it is worth the time and effort to make an experimental determination by X-ray crystallography.

**Fig. 8:**
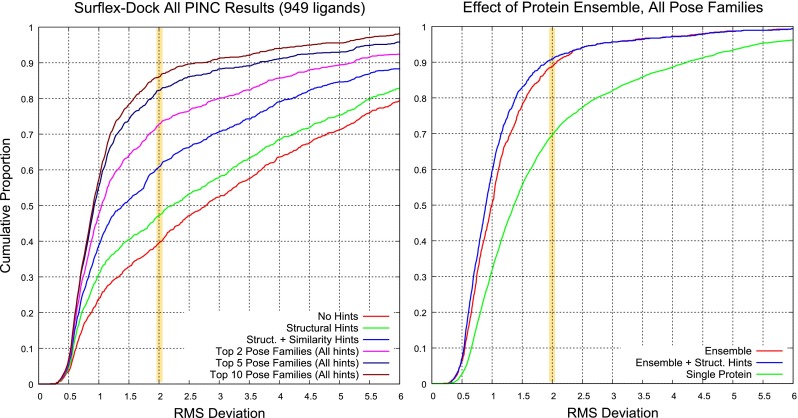
Overall performance of Surflex-Dock under different docking protocols (*left*) and considering the effect of using a protein ensemble (*right*) compared with a single protein variant for each target (aggregated over five different selections each)

Table 2 summarizes the prediction results for each target and overall. By combining substructural guidance during docking and similarity-based guidance in ranking pose families, we achieved a mean success rate for top-scoring pose families of 62 %. Considering the top two pose families, the overall success rate was 74 %. Figure 8 (left plot) shows the cumulative histograms of success rates aggregated for all targets under three protocols: (1) no knowledge-based guidance; (2) guidance only from substructural hints during docking; (3) and additional guidance from knowledge of bound ligands for pose family ranking (as with the results in Table 2). Each of the shifts in distribution of RMSD was statistically significant (*p*$\ll$ 0.001 by Kolmogorov-Smirnov).

**Table 2:** Summary of results in terms of success percentages at an RMSD threshold of 2.0 Å  for the top pose family, top 2, 5, and 10

Target	N test	Top family	Top 2	Top 5	Top 10
PTP1b	52	50	67	77	83
BACE1	103	57	71	85	88
MAPK14	92	71	79	89	93
HIV-PR	127	55	69	75	80
Thrombin	114	68	76	81	83
$\hbox {PPAR}\gamma$	62	31	34	39	45
CA-II	128	63	77	88	94
$\hbox {HSP90}\alpha$	57	57	72	91	91
CDK2	127	75	85	93	96
HIV-RT	46	63	78	87	91
Overall	949	62	74	83	87

At right in Fig. 8, performance is shown for *all* pose families to illustrate the effects of using protein ensembles. The red and blue curves show the effect of substructural guidance during docking, which is marginal at the 2.0 Å threshold, but significant overall. The green curve shows the effect of using the individual protein exemplars singly for all targets, giving the overall performance for all targets and all protein exemplars. The improvement using protein ensembles was highly significant compared with using single protein variants. The per-target patterns of performance using different single variants compared with using the ensemble exhibited some diversity, and this will be discussed below.

**Fig. 9:**
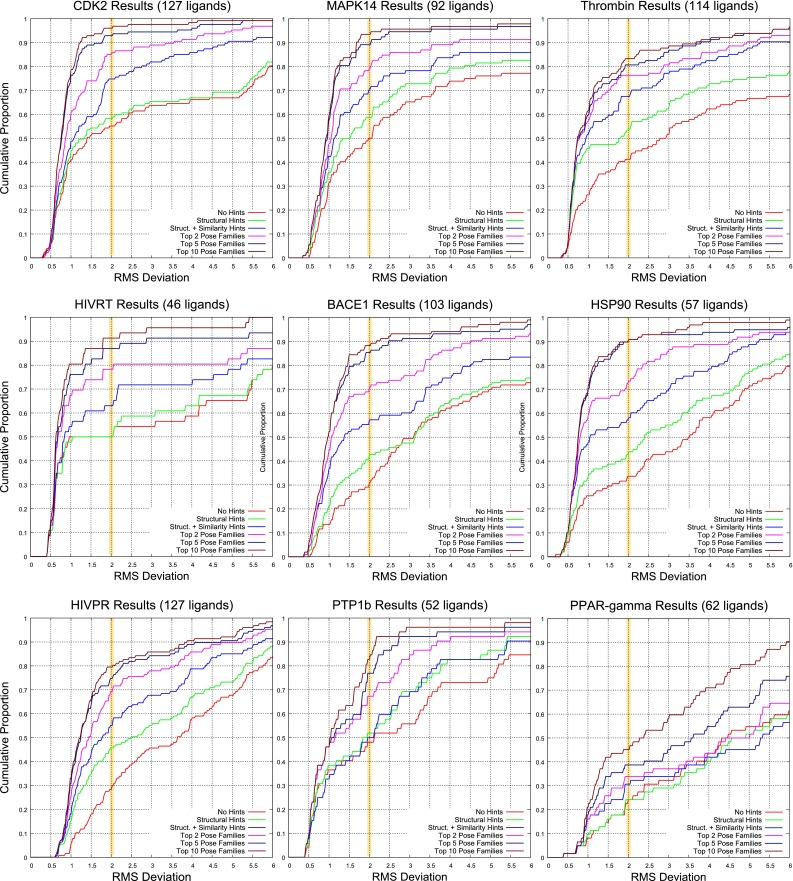
Overall docking performance under different docking protocols for nine targets. The key curves are blue (top scoring pose family using the knowledge-guided protocol), magenta (top two pose families), and red (top pose family in the unguided protocol)

Analogous plots for individual targets (all except for CA-II, which exhibited little relative novelty in the context of the other nine targets) are shown in Figs. 9 (overall docking performance) and 10 (effects of protein ensemble use). In Fig. 9, the blue and magenta curves correspond to the top scoring pose family and top two, respectively, with the yellow highlight bar showing the success rate for a threshold of 2.0 Å. When using both types of knowledge-based guidance, except for $\hbox {PPAR}\gamma$, typical success rates for top-scoring pose family (the blue curves) ranged from 60 to 75 %. The gap between the red and green curves represents the value of using substructural hints *during* the docking process. The gap between the green and blue curves shows the value of using similarity to known bound poses of ligands in addition to the substructural hints during docking. Overall, as seen in Fig. 8, the value of substructural hints was less (often substantially so) compared to similarity-based information for pose family re-ranking.

**Fig. 10:**
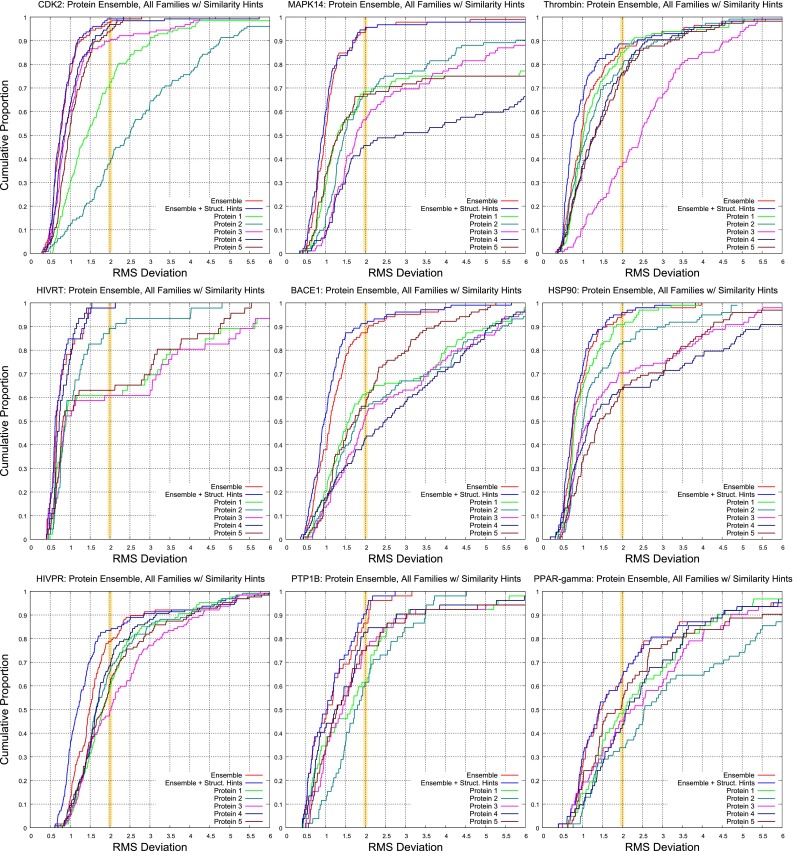
The effects of using single protein exemplars versus an ensemble of five for nine targets. The key comparisons are between the red curve (protein ensemble with no substructural guidance during docking), blue curve (adding substructural guidance), and the remaining curves (each from a single protein variant from the ensemble, using no substructural guidance)

The effects of using a protein ensemble versus using the individual exemplars within the ensemble were more varied. In all cases, it was possible to choose a particular protein from among each set of five that would produce substantially poorer performance than other variants or the ensemble produced. In a few cases, fortunate choice of a particular ensemble member yielded performance nearly as good as seen from the ensemble (see the thrombin and HSP90 examples, in particular). In two cases (MAPK14 and BACE1), performance using any single protein variant was *substantially* worse than that observed using the ensemble.

The three most challenging targets from our previous study (CDK2, thrombin, and MAPK14 [31]) yielded excellent performance in the current study, with mean performance of 71 % for top-scoring pose family and 80 % for the top two. In this study, the most challenging targets were $\hbox {PPAR}\gamma$, PTP1b, and HIV-PR. These shared in common the highest proportion of test ligands whose structures were not only very different by 2D similarity to previously known ligands, but they were also very different in terms of their maximal 3D similarity (in their bound pose) to previous ligands. Other aspects such as ligand size/flexibility and binding site volume were less important.

In what follows, performance on each target will be discussed in some detail, with particular attention paid whether the knowledge-utilization strategies had different impact on different targets. The order in which the discussion is organized is from best to worst performance for top-scoring pose family (using both types of structural guidance): CDK2, MAPK14, Thrombin, HIV-RT, CA-II, BACE1, $\hbox {HSP90}\alpha$, HIV-PR, PTP1b, and $\hbox {PPAR}\gamma$. This corresponds to the order seen in Figs. 9 and 10. Following that, the effects of our protein selection procedure will be discussed.

### CDK2

CDK2 was used as an example throughout the description of the methodology, as it represents a typical case in terms of performance within the overall benchmark. As seen in Fig. 9, the use of substructural information during docking produced a mild performance improvement (roughly 4 % points), but the use of similarity-based knowledge of prior known ligand binding modes produced a large improvement (roughly 17 points).

The reasons are two-fold. First, except for a few target cases, the search algorithms within the Surflex-Dock optimization procedure are generally adequate for identifying *some* close-to-correct poses within the set of 100 produced, even without using any information about previously identified binding motifs (the red curves in Fig. 10). Second, the difference in energy between native-like solutions (e.g. Fig. 7a) and incorrect ones (Fig. 7b) is small, often (as in this case) less than 1 kcal/mol. So the problem, generally speaking, is not one of discovering a good solution, but ranking it as such.

These observations paralleled our previous study, using a much smaller data set from Sutherland et al. [29]. In that work, CDK2 was among eight different targets, with 211 ligands used for testing overall. Pose ranking was the key problem, with less than half of the cases in which a correct pose was produced being correctly identified as such. In the previous work, protein pocket adaptation (Cartesian-space all-atom optimization) was used to influence pose family rankings. In the most difficult cases, of which CDK2 was one, such optimization was able to improve success rates for top-ranked pose family by a few percentage points. However, the case in which two variant methods for pocket adaptation resulted in *agreement* between the top-ranked pose families, there was a very substantial improvement in success rate.

In the present work, rather than identifying cases in which alternate methods agree on a particular ligand (which may happen infrequently), the current approach looks for agreement, measured by similarity, to the known configurations of bound ligands whose structure was determined at an earlier time point. For CDK2, this resulted in a success rate of 75 % for top-ranked pose family and over 85 % for the top two. Failures to identify any correct solutions happened less than 5 % of the time.

The behavior of the CDK2 case with respect to protein variant choice was also typical (see Fig. 10, top left). Two particular variants, when used alone (the green and teal curves), yielded poor performance. That is, even when selecting from among the five variants, each of which was itself the center of a cluster of variants, it was possible to obtain one that could not be used to identify reasonable poses for many ligands. Three other variants (the magenta, brown, and dark blue curves) performed nearly as well as the ensemble (red curve), except at lower thresholds of RMSD, where a significant advantage for the ensemble approach emerged. Importantly, it is not clear that one can know whether a single variant can be generally successful nor if that is the case which one will perform well. So, making use of the ensemble is an effective strategy. In some cases (discussed more below), it is essential.

**Fig. 11:**
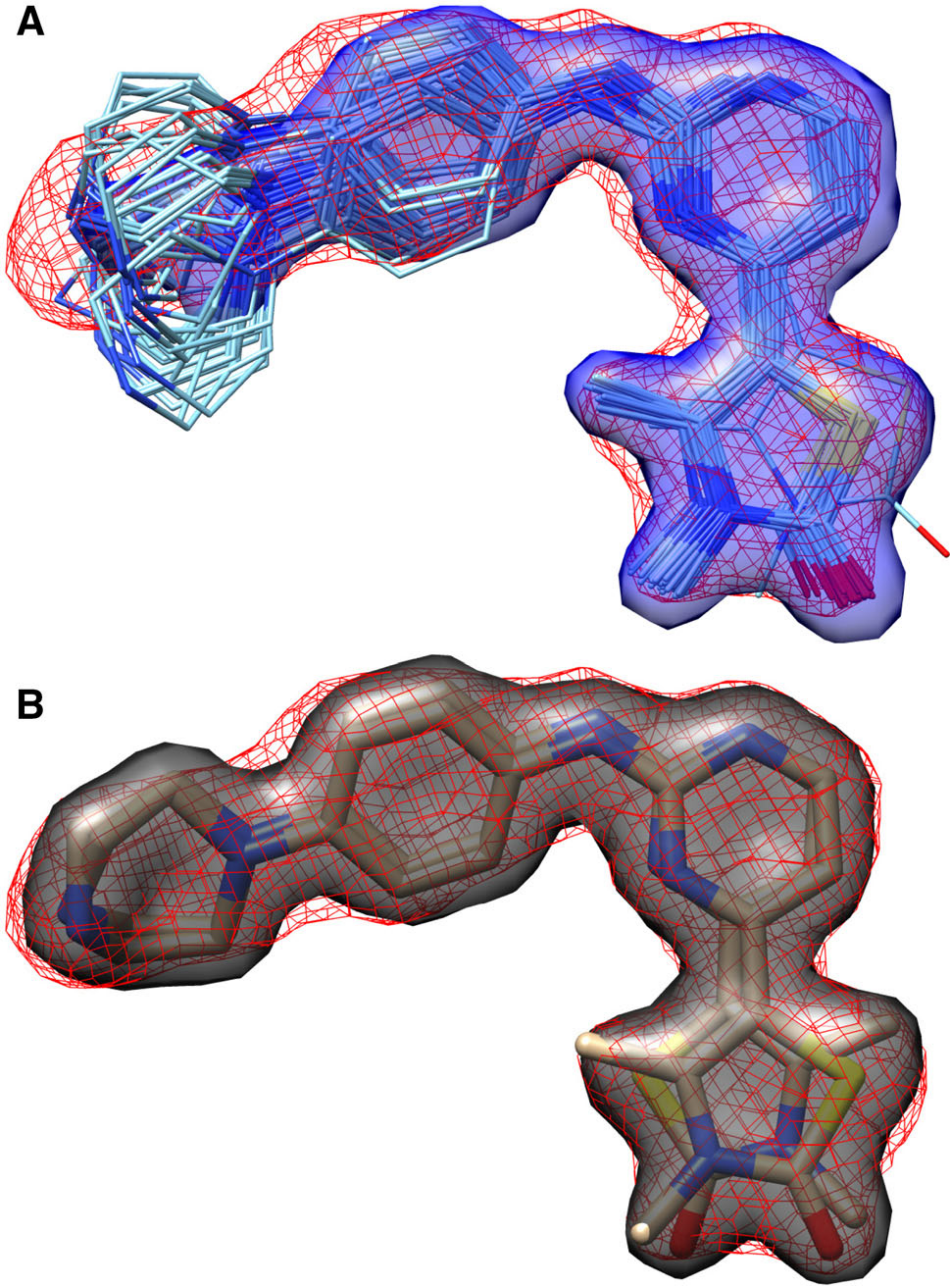
The experimental electron density (*red mesh*) for the 2XNB ligand is shown along with that computed for the entire top-scoring pose family ensemble (*thin cyan sticks* with *blue* transparent surface, **a**) and for the two alternate poses modeled in the crystallographic experiment (*thick tan sticks* with *gray* transparent surface, **b**)

One additional point is illustrated in Fig. 11. In the case of the 2XNB complex, the deposited structure contained two alternative poses for the ligand, the first of which was used for the deviation calculations. The bottom of Fig. 11 shows the density corresponding to the ligand (red mesh) along with the two modeled alternative poses, which appear to represent a good explanation of the observed density. The top of the figure shows the full set of poses for the top-ranked pose family, along with an imputed density surface (in transparent blue), contoured to provide a comparison with the experimental density. It seems probable that numerous solutions exist which simultaneously respect the internal energetics of the ligand, the observed density, and sensible interactions with the protein. While the positional variation seen at the left-hand-side of the ligand in the full predicted pose family may extend beyond what is supportable by experiment, more variation than is represented in the modeled structure may exist. We believe that the perspectives of both the structural biologist and the molecular modeler can benefit from broader consideration of pose variants than has been historically done.

#### MAPK14

Overall performance on MAPK14 was very similar to that seen for CDK2, with the exception that the benefit from making use of substructural knowledge was slightly higher. There was a roughly 9-point difference in success rates a the 2.0 Å threshold when comparing the red and green cumulative histograms of Fig. 9. The top scoring pose family using both types of knowledge-based guidance was 70 %, and the top two pose families yielded 80 % correct. Note that, as with CDK2, MAPK14 was among the three most challenging targets in our previous work, but the combination of knowledge-guidance from substructural hints and similarity-based pose family re-ranking made it the second-best in this work.

As with CDK2, there was significant performance variation among the five chosen protein pocket variants, when used singly (see Fig. 10, top middle plot). However, in sharp contrast, the very best of these performed 26 % points worse than the ensemble. In this case, joint use of the five proteins was crucial to uncovering correct solutions for many ligands. The only other target where this pattern emerged was BACE1. These two cases were used for a systematic test of different strategies for choosing protein ensembles, the results of which will be discussed after the individual protein targets.

#### Thrombin

**Fig. 12:**
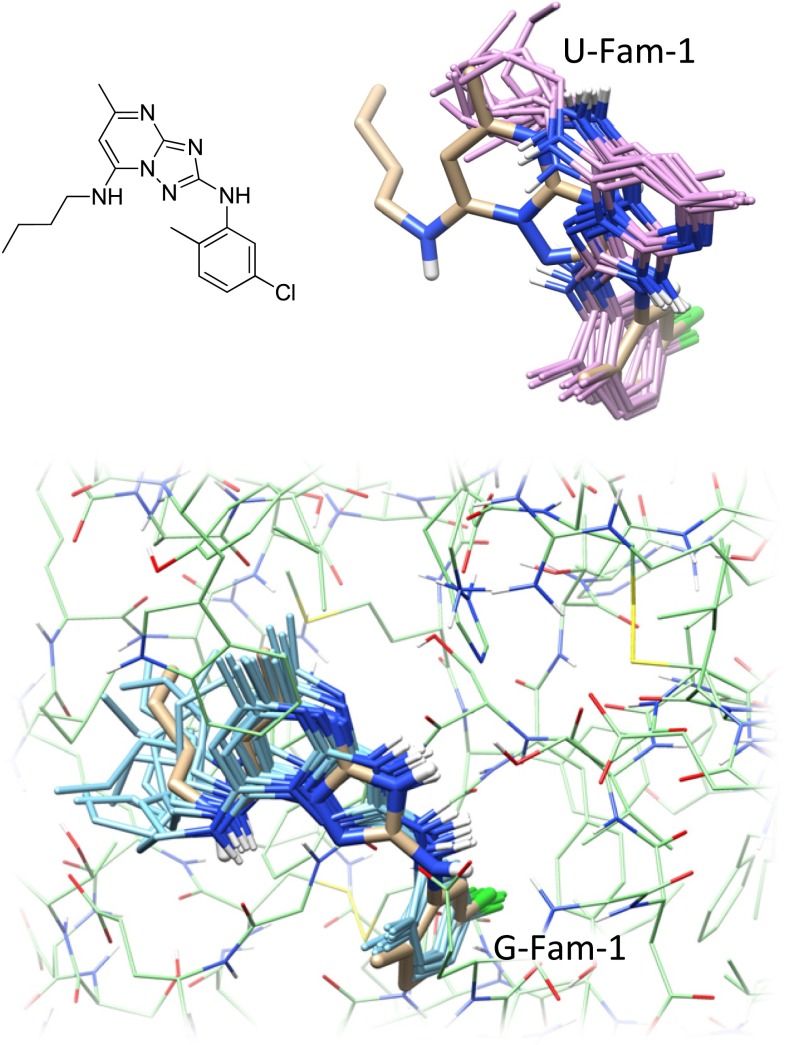
For thrombin, comparison between docking without knowledge-based guidance (*top right*, *pink*) and with guidance (*bottom*, *cyan*) for the ligand of 1ZGV, a triazolo-pyrimidine with a non-basic S1 binding element, (*top left* in 2D and thick tan sticks in its experimental pose)

Thrombin was the third of the three targets shared with our previous cross-docking study, and as with the previous two discussed, was one of the most challenging. In that work, top-scoring pose family success was roughly 50 %, with the top two pose families achieving roughly 60 %. Here, the comparable numbers were 68 and 76 %, with the difference being essentially entirely attributable to the use of similarity-based re-ranking of poses. Figure 12 shows the results of docking for the test ligand from PDB structure 1ZGV, an example of a thrombin inhibitor with a non-basic S1 binding pocket element. The result obtained from an agnostic docking protocol yielded an RMSD of 3.7 Å (top), getting the placement of the S1-pocket element correct, but flipping the remainder of the molecule out of the correct pose. The unguided protocol contained an excellent solution (0.7 Å), but the docking score for that solution did not result in the top-scoring pose family. Use of substructural guidance produced more numerous and better solutions, which, with the inclusion of similarity-based re-ranking, yielded a solution with just 0.6 Å deviation (bottom of Fig. 12). This case represented a thrombin inhibitor of limited flexibility (just 6 rotatable bonds), which presented little challenge with respect to search adequacy but was difficult in terms of the precise ranking among the poses produced.

**Fig. 13:**
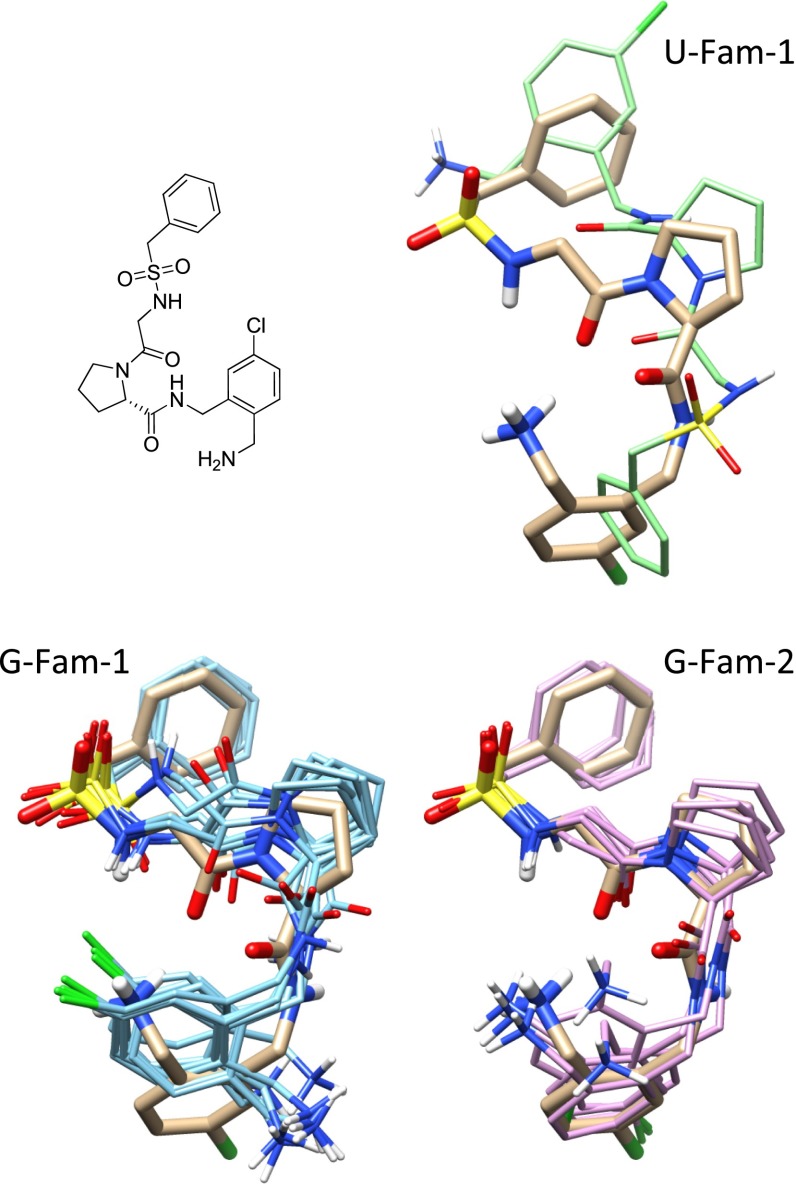
Comparison between without knowledge-based guidance (*top*) and with guidance (*bottom*) for the thrombin ligand within 3RML, showing the top two pose families in the guided case

A more challenging example, with 11 rotatable bonds, is shown in Fig. 13. This structure was deposited in the PDB in April 2011, nearly ten years after the most recent structures from the “known” pool. The top-scoring pose family in the agnostic protocol contained a single pose, which was flipped completely around the central proline, resulting in a deviation of 8.5 Å. Under the guided protocol, the top-scoring family (bottom left), achieved a degree of congruence with the experimental solution, correctly placing the sulfonamide substituent and obtaining grossly correct positions for the proline linker and the chloro-benzylamine (2.3 Å RMSD, 51 % probability score). The second pose family (46 % probability) was correct, deviating 0.5 Å from the experimental pose.

The nominal docking scores of the various solutions represented in Fig. 13 were within 1.0 kcal/mol of one-another. Cases such as this, with flexible peptide-like ligands having low ligand-efficiency, are among the most challenging in pose prediction. Approaches that seek to disambiguate such pose variants using purely energetic estimation approaches face a high bar. Note that the correct position of the primary amine is *away* from the aspartic acid residue within the S1 pocket of thrombin, instead being apparently stabilized through intramolecular contacts. Note also that the early complexes used to inform the docking and pose-ranking protocol were dominated by basic groups at the S1 position, with no examples of chloro-phenyl or similar groups. The binding motif seen in the linker from the S1 binding element (including the sulfonamide) to the hydrophobic substituent was of use in identifying the correct configuration.

#### HIV-RT

The HIV-RT ligands were bound in the non-nucleoside binding site, which was the smallest site, by far, among the ten targets studied (the next larger site of CDK2 was slightly more than twice the volume). This, coupled with limited ligand flexibility (an average of 5 rotatable bonds), mooted the issue of search adequacy. No improvement was observed using substructural hints during docking (see Fig. 9, left side, middle plot). In fact, over 95 % of test ligands yielded a predicted pose with deviation less than 1.5 Å from experimental when considering the full set of pose families produced (see Fig. 10). As was typical, particular choices of protein variant could yield poor results. However, in this case, there was a single pocket variant (protein 4) that performed indistinguishably from the ensemble. HIV-RT was the only example where this was clearly the case.

Despite the small volume, pose ranking for the small, hydrophobic ligands was a challenge. Top-scoring pose family performance was 63 %, with performance improving to 78 % when considering the top two families. Even when considering the top ten families, performance was 91 %, still less than the 98 % success attainable (all but 1 of the 46 test ligands) when considering all pose families that were generated.

**Fig. 14:**
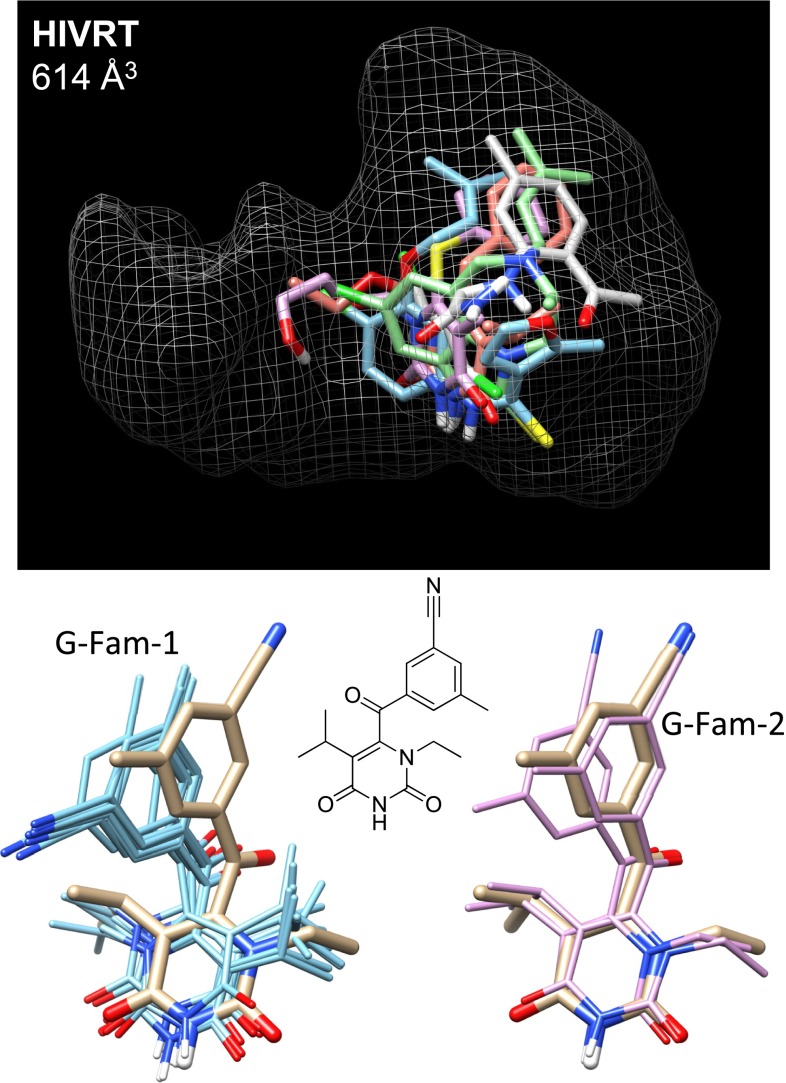
The HIV-RT pocket volume (*top*) is shown along with docking results for the ligand of 3LAL: *tan sticks* for the experimental pose, *cyan* for the top-scoring pose family with knowledge-based guidance, and pink for the second-ranked family

Figure 14 shows the pocket volume along with docking results that illustrate the challenge within this small pocket. The top-scoring pose family (bottom left, cyan) deviated by 4.0 Å from experimental. The top-scoring family from the unguided protocol was worse still (not shown). Clearly, the second-ranked pose family (pink) matches the experimental pose better (0.5 Å RMSD), but the nominally large difference in deviation between the two alternative stems from two reasonable “flips.” The pyrimidine-dione is flipped in the top-ranked configuration (“G-Fam-1”), placing the N-ethyl at right, but the core scaffold is nearly symmetric. The methyl and nitrile substituents are also reversed, again not unreasonably. This is a case where nominal RMSD gives an incomplete picture of how informative a geometric prediction may be.

#### BACE1

Apart from being a more challenging case, BACE1 exhibited the same overall pattern as MAPK14 in terms of the performance benefits of knowledge-based guidance and sensitivity to use of pocket variants compared with the full ensemble. Substructural guidance during docking provided a roughly 12-point improvement in performance (Fig. 9), and similarity-based re-ranking produced roughly 17 points on top of that, resulting in top-scoring pose family performance of 57 %. Performance improved to 71 and 85 % when considering the top two and five pose families, respectively. The ensemble approach produced a more than 25-point improvement over the next best single protein variant (Fig. 10). The effect of protein variant selection strategy for BACE1 will be discussed below.

Note that BACE1 had the second-most flexible set of test ligands (next to HIV-PR), with mean flexibility of 10 rotatable bonds ($\pm 7$). It was also the second largest site by volume (next to PTP1b), with the site enveloping 2360 Å$^3$. BACE1 is generally considered to be a challenging target, in part because of the size and flexibility considerations, so the pose-prediction performance we observed was striking. Here, top-ranked pose family performance on diverse and highly flexible ligands in a temporally segregated *cross-docking* test matched that observed on challenging *cognate* docking benchmarks [13, 14, 23] for multiple docking methods (including methods such as Glide, ICM, GOLD, and Surflex-Dock).

#### HSP90

The pattern of performance improvements for HSP90 most closely paralleled that of CDK2, albeit at lower levels of overall success. Substructural guidance during docking provided a roughly 10-point improvement in performance (Fig. 9), and similarity-based re-ranking produced roughly 13 points on top of that, resulting in top-scoring pose family performance of 57 %. Performance improved to 72 and 91 % when considering the top two and five pose families, respectively. The ensemble approach produced a 5-point improvement over the best single protein variant (Fig. 10), and it was roughly 30 points better than the worst variant.

#### HIV-PR

HIV-PR was, by a significant margin, the target with the most flexible ligands (an average of $16\pm 5$ rotatable bonds). That fact, coupled with an active site volume of nearly 2000 Å$^3$, and a reasonably flexible protein, created an a priori expectation of high difficulty. It was atypical in that it was the only target for which substructural guidance during the docking process yielded a larger improvement (15 points) than similarity-based re-ranking (10 additional points). This was likely due to the extreme flexibility of the test ligands. Top-scoring pose family performance was 55 %, increasing to 69 % for two, and 75 % for top five. The value of substructural hints is clearly seen in Fig. 10 (bottom left plot), where the difference between the red and blue curves is only the use of substructural guidance. At the 2.0 Å threshold, such guidance yields a six-point advantage (for an overall success rate for all pose families of 84 %). At the 1.5 Å threshold, the improvement was 20 points; clearly a very significant impact. Similar to BACE1 and MAPK14, but to a lesser degree, the use of a protein ensemble produced better results than any single protein variant.

**Fig. 15:**
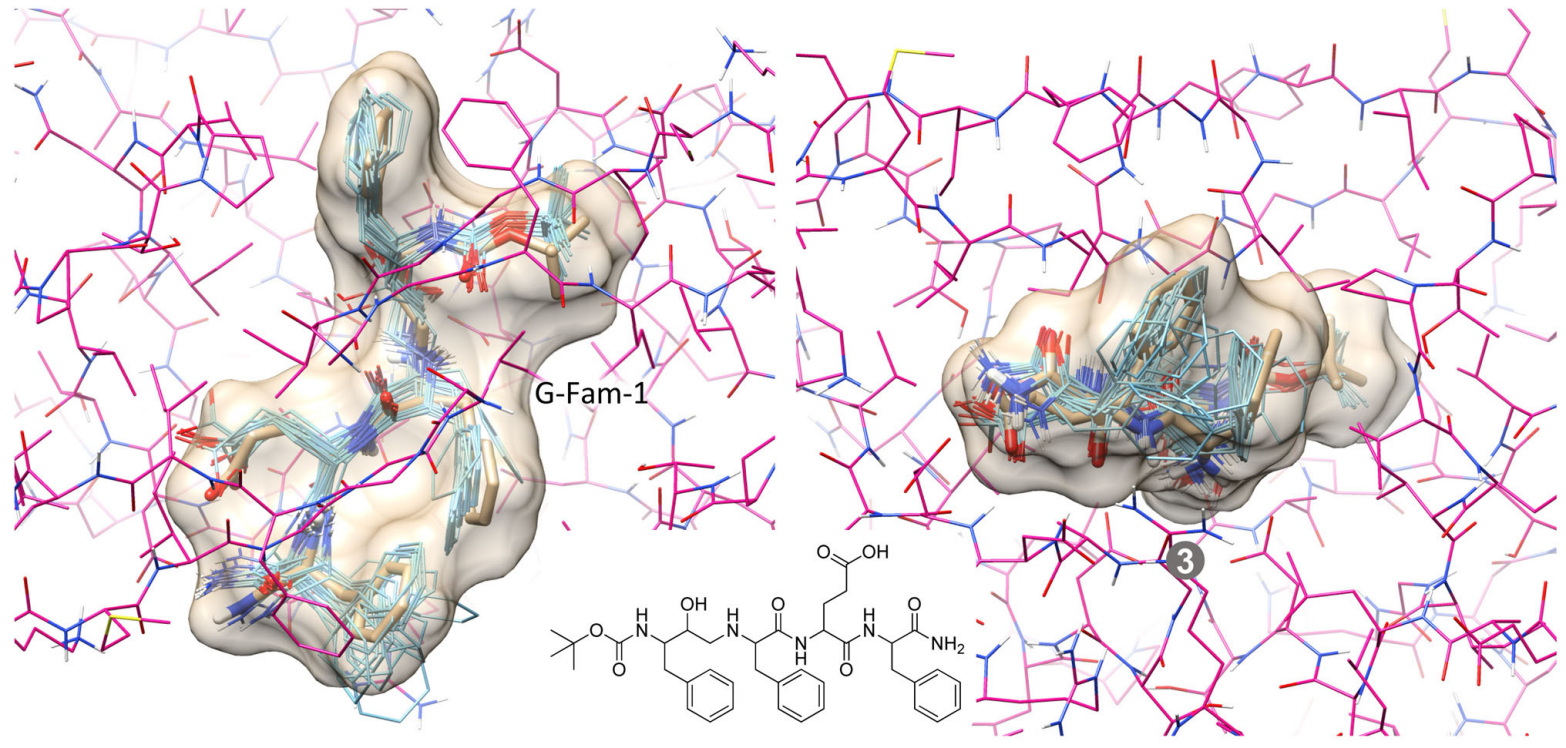
HIV-PR (*magenta*), shown in top view (*left*) and side view (*right*), with the top-scoring predicted pose family for the ligand of 1ZSR (*cyan* with experimental pose in *tan*)

Figure 15 shows a typical peptide-like inhibitor (the ligand of 1ZSR), having over 20 rotatable bonds. Two views are shown of the top-scoring knowledge-guided pose family, with the crystallographic pose shown in tan and with a transparent surface. The RMSD of the closest pose within the family was 0.8 Å, and as the inhibitor meets solvent (bottom left and front right in the figure) it exhibits a greater degree of mobility in the docking result. We believe that this picture of a binding-mode prediction is more informative, and likely more accurate, than one where a single pose is displayed.

Especially given the demanding characteristics of HIV-PR, we view the performance of the knowledge-guided protocol as being a success. More broadly, to summarize thus far, performance using the knowledge-guided protocol for all but two of the ten targets reported here (PTP1b and $\hbox {PPAR}\gamma$, discussed below) met or exceeded 55 % for top-ranked pose family and all but one ($\hbox {PPAR}\gamma$) met or exceeded 67 % for the top two. Except for perhaps two targets, the level of performance seen here for cross-docking matches that of challenging cognate docking benchmarks. It significantly exceeds that previously reported on substantial cross-docking benchmarks such as those described in the Introduction, where success rates of 20–30 % were common in cross-docking with single protein variants [28–31]. Note also that this is the only temporally segregated benchmark of which we are aware, and it is also one of the largest and most diverse in terms of both target types and ligand structural variety.

#### PTP1b

**Fig. 16:**
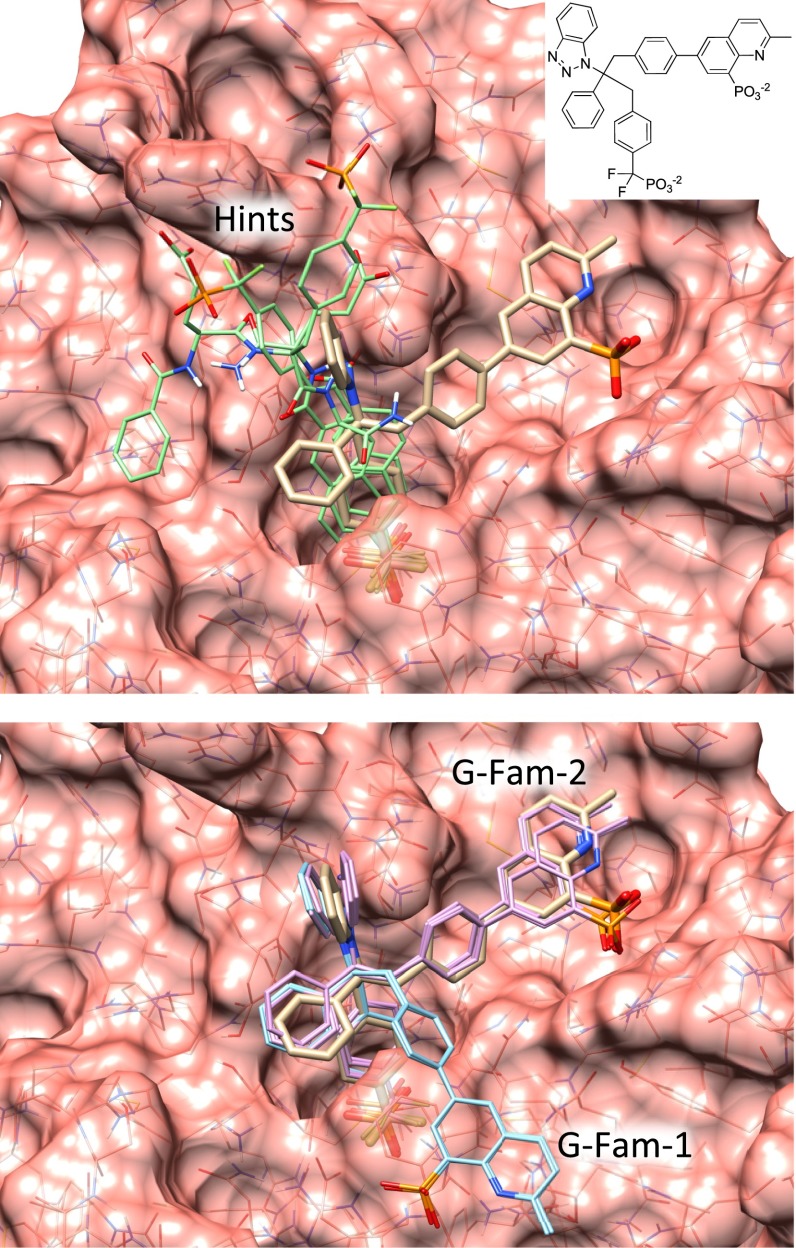
PTP1b (*pink*) is shown with the ligand of 1Q6S (*tan sticks*), the relevant known molecules sharing the difuoromethylphosphate (*green*), and the top two predicted pose families (*cyan* and *light magenta*)

PTP1b had the largest active site (over 3000 Å$^3$), and its ligands were quite flexible (an average of $10\pm 5$ rotatable bonds). The unusual aspect of performance for this target was that, at the 2.0 Å threshold, no real improvement resulted from use of either method for making use of prior knowledge, at least for top-scoring pose family. At larger deviations, there was a clear benefit for using substructural guidance (see Fig. 9), but not for similarity-based re-ranking. PTP1b also had the largest increase in success in moving from a single pose family to two (17 % points). Figure 16 illustrates the challenge of this binding site with an example of the improvement seen between the top and next best scoring pose family under the guided protocol. The top scoring pose family, despite having placed the buried substituent correctly, places the rigid “arm” of the inhibitor in an incorrect position along the surface of the protein (7.9 Å RMSD). The second-ranked pose family (0.5 Å RMSD) was correct. The nature of binding for large inhibitors in this class is mainly on the protein surface, where much less physical constraint exists to constrain potential docking solutions.

#### $\hbox {PPAR}\gamma$

$\hbox {PPAR}\gamma$ was an outlier in terms of performance under all circumstances: with and without knowledge-based guidance and using any number of top-scoring pose families. For the other nine targets, using the knowledge-guided protocol, the success rate at the 2.0 Å threshold was $0.62\pm 0.08$ for the top-scoring pose family. For the top two, it was $0.75\pm 0.06$, and for the top five, it was $0.85\pm 0.06$. Performance for $\hbox {PPAR}\gamma$ was, respectively, 31, 34, and 39 % under the same docking protocol, representing decreases in performance of $4\sigma$ or greater.

The active site of $\hbox {PPAR}\gamma$ was of moderate volume (1400 Å$^3$) and the ligands were of moderate flexibility (an average of $8\pm 4$ rotatable bonds) for this set of targets. The reasons for the difficulty appear to stem from two primary drivers: (1) diversity in protein active site configurations when bound to the test ligands; and (2) the fraction of novel binding modes of test ligands compared with training ligands.

For $\hbox {PPAR}\gamma$, protein binding site diversity was the highest among all proteins. We computed the maximal binding pocket similarities for each cognate pocket for each test ligand against the pockets for all ligands within the known early pool. We assessed the fraction of such similarities that fell below a threshold set based upon a global analysis of computations for all proteins (see Methods), calling those sites with lower similarity novel. For $\hbox {PPAR}\gamma$, the fraction of novel protein active sites was 49%. Interestingly, the two targets that benefited the most from making use of a protein ensemble (MAPK14 and BACE1) also had high novelty fractions (33 and 18 % respectively). Among the remaining targets, active site novelty fractions were all below 10 %, except for HIV-RT (22 %) whose limited volume appears to ameliorate that effect.

To assess novelty with respect to test ligand binding mode, we performed a similar computation using 3D similarity of the bound configurations of test ligands compared with those of the known early pool. For each test ligand, the maximum similarity to the knowns was computed. Novel binding modes accounted for fully 50 % of the ligands for $\hbox {PPAR}\gamma$. This appears to explain the relative challenge of PTP1b as well, with 40 % of test ligands exhibiting novel binding modes. The remaining targets exhibited novel binding modes less than 10 % of the time, except for HIV-PR (28 %) which was also a relatively challenging target.

**Fig. 17:**
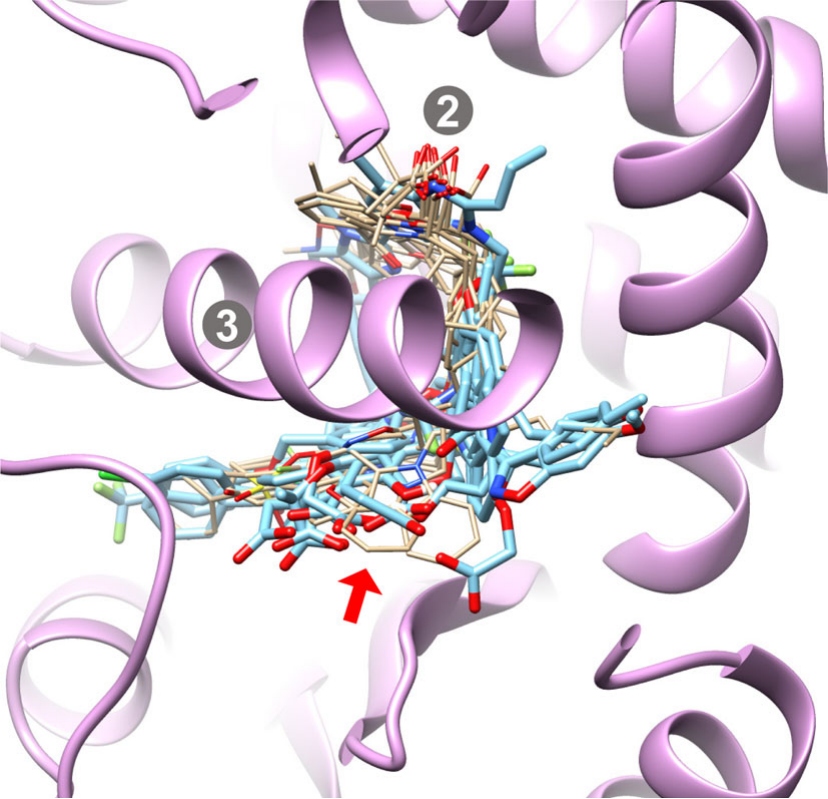
Canonical early bound ligands of $\hbox {PPAR}\gamma$ with a shared binding mode (*tan*), along with the nine worst test cases (*cyan*), all exhibiting a completely different binding mode for organic acids

To put this issue of binding-mode novelty in perspective, recall Fig. 3. The marked locations “2” and “3” correspond to those marked in Fig. 3: (1) the canonical binding location for acids; and (2) helix 12, the canonical helix around which many ligands are known to bind [42]. Location 2 is surrounded by four donor protons (two from histidine residues, one from serine, and one from tyrosine). Figure 17 shows 11 canonical ligands (tan) from within the early pool of 21 complexes that contained carboxylates in favorable contact with this part of the protein. Also shown are the 9 worst failures (cyan, based on best RMSD among the top ten pose families). All of the latter placed carboxylates in a completely different place than that seen in the canonical binding mode. In addition, a critical arginine residue moves several Angstroms in order to complement the binding mode seen for these difficult test ligands.

The extreme difficulty of this target was well documented in a structural sense by Itoh et al. [42], where hydroxyoctadecadienoic acid variants were shown to be capable of binding in three divergent modes to $\hbox {PPAR}\gamma$. One mode was in the canonical position, another in the alternative mode that was common among the docking failures, and a third in which a second ligand could bind at the same time as one binding in the canonical mode (where there was also a critical contact made between the ligands). The $\hbox {PPAR}\gamma$ case represents a true limitation for the methods described here. Binding modes that are completely unlike those seen earlier will not be recovered through use knowledge from previous ligands, either in terms of substructural matching for configurational search or similarity-based pose re-ranking. Further, careful automated choice of protein pocket variants from among a set that *does not* contain a crucial rearrangement cannot help to identify novel binding modes as are seen with this target.

### Effect of protein variant selection

As seen in Fig. 10, selection of protein variants matters in all cases, at least to the extent that a poor choice of a single variant could lead to significantly worse results than the choice of an optimal variant. This was also true for CA-II (whose plot is not shown), especially at more stringent levels of RMS deviation. In all cases, performance of the ensemble (red curves) was much better than the worst single variant, and in no case was the ensemble worse than the best of the single variants. In two cases, MAPK14 and BACE1, the ensemble was more than 25 points better than that seen with the *best* single protein variant. This was, in part, explained by the analysis of protein pocket novelty, with these two cases showing a relatively high fraction of novel pocket variations among the test complexes.

In order to assess the degree to which the protein variant selection strategy was successful, two additional methods for selecting five variants were tried for MAPK14 and BACE1. The first was a maximally diverse selection strategy. Recall that the strategy described earlier made use of K-means clustering (with K of 5) along with selection of a particular variant for each cluster whose average similarity to the other members was highest. For the maximally diverse choice, the set of 5 proteins that were maximally dissimilar to one another were chosen (using a greedy algorithm beginning with the single protein pocket most dissimilar from all others). The second was a purely random strategy, in which five different sets of five were randomly chosen.

Performance was assessed using the protocol without any knowledge guidance when considering all protein families generated from docking. For the K-means strategy used throughout the paper, the success rates were 95 % for MAPK14 and 87 % for BACE1. The “diverse” strategy success rates of 66 and 82 %, respectively. For MAPK14, the drop of 29 points was highly statistically significant (*p*$< 10^{-6}$ by exact binomial). For BACE1, the drop of 5 points was just significant at the *p* = 0.05 level.

Using random selections, the average performance for MAPK14 was $82\,\%\pm 14$. Two of the five random selections performed as well as the K-means strategy (success rates of 96 and 95 %), but three were significantly worse. For BACE1, the average performance of the random selections was $85\,\%\pm 2$, matching that of the K-means approach.

The K-means approach was at least as good as the best of any alternative selection method, and it was clearly superior to the “diverse” approach. The latter essentially identifies outliers in protein pocket conformational space, which probably do not represent the bulk of relevant configurations for predicting the binding modes of new ligands. Perhaps surprisingly, choosing random sets of 5 proteins each was a better approach than choosing maximally different variants. In fact, for MAPK14, a fortunate choice of variants was as good as the careful K-means approach in 2/5 replications. For BACE1, the random approach never performed better than the K-means approach, but it did not perform worse either. Overall, the evidence suggests that making use of the K-means approach will result in the best performance, but testing such strategies on additional targets appears warranted.

## Conclusions

We have presented a new benchmarking data set for assessing pose prediction using molecular docking called PINC. The benchmark is focused on targets of pharmaceutical interest (ten total), where, for each target there is an average of 95 ligands for testing (minimum of 46 and maximum of 128). The test ligands were partitioned from the training ligands temporally, with the *earliest* 25 % of complexes deposited in the PDB being used as information for use in making predictions on the remaining 75 %. This was done for two reasons. First, it is likely that new complexes are sought in cases where uncertainty exists as to either the binding mode of a ligand or its effect on the protein conformation. Second, it has been well established that random partitioning has significant liabilities in assessing the performance of predictive modeling for drug design, because ligands are the products of human invention, with their structures often reflecting their ancestry [24, 37, 40, 43, 44]. So, making use of a “future” ligand in order to predict the binding mode of a “past” one can often embed the correct answer within the prediction task.

No effort was made to adjust the proteins used for docking to suit the test ligands (either by selection or by modification). Also, none was made to adjust test ligands protonation or tautomeric state to match that of the proteins used for docking (the test ligands were prepared with reference to their cognate proteins only). Last, no quality parameters were used to limit the set of complexes that formed the benchmark. Altogether, we believe the PINC set to be the most relevant to the real-world problem of structure-based pose prediction for small-molecule ligands that exists.

We have presented two algorithmic enhancements to Surflex-Dock, both allowing for exploitation of knowledge of ligands whose binding mode had been previously determined. The first, used during docking, automatically identifies relevant matching subfragments between a subject ligand and known ones in order to focus additional search on binding modes that have been seen previously. The benefit of this method was typically 5–10 % points in terms of improvement to top-scoring solutions at the 2.0 Å RMSD success threshold, but it was larger than that for targets with particularly flexible ligands.

The second enhancement made use of an idea from statistical physics, where the degree to which a predicted pose looked “native-like” was used in order to adjust its docking score. A pose whose 3D similarity was unusually high to those of known ligands in their experimentally determined binding modes is quantitatively adjusted in energy based on the estimated ratio of native-like to non-native probability. This similarity-based re-ranking of poses yielded 10–20 points of improvement in success rates.

We also made used of a careful strategy to choose among available protein variants using protein binding pocket similarity [27, 33, 34, 45]. The strategically chosen ensembles always performed as well as the best of all alternative selection strategies, occasionally providing as much as 25-point improvements over the best single protein variants and always providing substantial benefits over the worst single variants.

All targets showed similar patterns of performance benefits, with ensemble docking using five automatically chosen protein variants, coupled with use of both types of knowledge-based guidance for pose prediction producing the best results. For nine targets (all but $\hbox {PPAR}\gamma$), performance for this very challenging cross-docking problem matched that seen for difficult cognate docking benchmarks. In particular, the success rate at the 2.0 Å threshold was $0.62\pm 0.08$ for the top-scoring pose family. For the top two, it was $0.75\pm 0.06$, and for the top five, it was $0.85\pm 0.06$. We believe that, in cases where there is binding mode uncertainty, manual visualization of a handful of possible solutions is something that most modelers will be willing to contemplate.

Clearly, there are many different strategies that could be explored to achieve performance benefits such as those we present here. In particular, choice of protein variant on a per-ligand basis, as opposed to the ligand-independent approach we have used, could be useful. Certainly, it has been established in earlier cross-docking studies that in cases where the ligand to be docked is highly similar to the cognate ligand of a particular crystal structure, the chances of successful pose predictions from docking increase [30]. In this benchmark, the fraction of cases where *any* of the known structures contain a ligand that is highly similar to one to be docked is relatively low, and it is the lowest for the most challenging targets. Likewise, there are many ways in which one can combine ligand similarity approaches with protein structural data. It is our hope that the public availability of this benchmark will help to both develop and evaluate such methods.

As discussed earlier, the performance reported here represents a significant improvement in a numerical sense over previous cross-docking studies (even those constructed with less challenging intentions). The importance of this improvement depends, to some degree, on how docking is used in practice. We have not addressed the virtual screening application of the methods described here; that will be something addressed in future work. The use-case considered here, prediction of bioactive poses, is relevant in at least two ways: (1) hypothesis generation for lead optimization; and (2) as a starting point for binding affinity prediction.

Often, docking is used as an informal adjunct to lead optimization, in which consideration of the likely binding mode of a ligand within an active site informs ideas about new molecules. Based on the temporal construction of the benchmark, it is likely that binding modes were in question for many of the cases examined here. We obtained prediction of correct binding modes within the top two solutions 75 % of the time for all but one of ten targets. Experienced modelers working on familiar proteins could achieve some of the benefits of the automated methods presented. We still believe that the lead optimization scenario is one where there is likely to be a practical impact for what we have reported here. When a new series is identified, either through screening or through public disclosure in the literature, rapid exploitation of the information is clearly valuable. The methods presented here make it possible to systematically exploit large quantities of pre-existing biophysical data.

Another related, but subtly different, use of docking is to build support for a modeler’s notion of how a ligand may bind. In such cases, direct “steering” of the docking method is used to decide whether any pose exists for the ligand that appears compatible with the hypothesis and with available structural data. The methods presented here offer an agnostic means to see if the modeler’s hypothesized binding mode is independently reproducible within a highly-ranked pose family. When confirmation exists, it should provide confidence in the hypothesized binding mode. However, the methods do not directly support such active steering for hypothesis verification. The methods are designed for automated use.

Perhaps more important, though, is that computational approaches for predicting binding affinity increasingly are dependent on close-to-correct relative (or absolute) binding configurations in order to produce the most accurate results. We have recently developed a structure-based means to influence the construction of physical binding site models for predicting ligand affinity [27]. In that approach, docking is used to help construct hypotheses for initial ligand alignments in order to bias model induction toward solutions that are closer to biological reality, and models constructed in such a manner are able to make accurate affinity predictions on a broad variety of new ligands. Methods utilizing molecular dynamics, such as MM/GBSA and MM/PBSA, *require* that accurate binding modes for all ligands be known [46–48], so the methods reported here could be utilized in such protocols as well.

Another observation through the course of this study has been that ligand binding modes are, nearly always, representable by quite large sets of closely-related poses. We believe that the picture that is promoted by looking at protein-ligand complexes as single, static configurations is inaccurate and that it limits creativity in thoughts about molecular design. We hope to develop methods to help identify ligand variations that better model the experimental data in X-ray crystallography. We also plan to make use of the concept of pose families and information fusion using probabilistic methods to improve both the quality and interpretability of 3D-QSAR methods.

The work presented here represents the first generalization of our ongoing work using such techniques for predicting polypharmacology [40, 41, 49]. We believe that hybrid approaches that combine information from docking and scoring, ligand similarity, and protein pocket similarity will frequently show synergistic performance improvements for lead discovery and for predictions of binding mode, affinity, and off-target biological effects.
